# c-Myc-driven glycolysis polarizes functional regulatory B cells that trigger pathogenic inflammatory responses

**DOI:** 10.1038/s41392-022-00948-6

**Published:** 2022-04-18

**Authors:** Xu-Yan Wang, Yuan Wei, Bo Hu, Yuan Liao, Xiaodong Wang, Wen-Hua Wan, Chun-Xiang Huang, Mahepali Mahabati, Zheng-Yu Liu, Jing-Rui Qu, Xiao-Dan Chen, Dong-Ping Chen, Dong-Ming Kuang, Xue-Hao Wang, Yun Chen

**Affiliations:** 1 MOE Key Laboratory of Gene Function and Regulation, Guangdong Province Key Laboratory of Pharmaceutical Functional Genes, School of Life Sciences, Sun Yat-sen University, Guangzhou, China; 2 Department of Laboratory Medicine, the Third Affiliated Hospital of Sun Yat-sen University, Guangzhou, China; 3 School of Pharmaceutical Sciences, Shenzhen University Health Science Center, Shenzhen, China; 4 Hepatobiliary Center, The First Affiliated Hospital of Nanjing Medical University, Key Laboratory of Liver Transplantation, Chinese Academy of Medical Sciences, NHC Key Laboratory of Living Donor Liver Transplantation (Nanjing Medical University), Nanjing, China; 5 Department of Immunology, Key Laboratory of Human Functional Genomics of Jiangsu Province, Nanjing Medical University, Nanjing, China; 6 Jiangsu Key Lab of Cancer Biomarkers, Prevention and Treatment, Collaborative Innovation Center for Cancer Personalized Medicine, Nanjing Medical University, Nanjing, China

**Keywords:** Inflammation, Lymphocytes

## Abstract

B cells secreting IL-10 functionally are recognized as functional regulatory B (B_reg_) cells; however, direct evidence concerning the phenotype, regulation, and functional and clinical relevance of IL-10-secreting B_reg_ cells in humans is still lacking. Here, we demonstrate that, although IL-10 itself is anti-inflammatory, IL-10^+^ functional B_reg_ cells in patients with systemic lupus erythematosus (SLE) display aggressive inflammatory features; these features shift their functions away from inducing CD8^+^ T cell tolerance and cause them to induce a pathogenic CD4^+^ T cell response. Functional B_reg_ cells polarized by environmental factors (e.g., CPG-DNA) or directly isolated from patients with SLE mainly exhibit a CD24^int^CD27^−^CD38^−^CD69^+/hi^ phenotype that is different from that of their precursors. Mechanistically, MAPK/ERK/P38-elicited sequential oncogenic c-Myc upregulation and enhanced glycolysis are necessary for the generation and functional maintenance of functional B_reg_ cells. Consistently, strategies that abrogate the activity of ERK, P38, c-Myc, and/or cell glycolysis can efficiently eliminate the pathogenic effects triggered by functional B_reg_ cells.

## Introduction

Regulatory B (B_reg_) cells are now recognized as suppressive cells that support immunological tolerance^1^. A shared signature of all described B_reg_ subsets is their ability to release IL-10, and in parallel, release of IL-10 is extensively used to define functional B_reg_ cells^2–4^. Nevertheless, in addition to producing IL-10, B_reg_ cells also express other molecules (e.g., PD-L1, FasL, and granzyme B), which induce additional pathological effects^5–7^. Thus, IL-10 is necessary, but not sufficient, for B_reg_ cell activities. Studies have established that immature transitional CD24^hi^CD38^hi^ B cells^8^, together with mature CD24^hi^CD27^+^ B cells^9^, represent important cellular sources of peripheral B_reg_ cells, both in healthy individuals and in patients. Of note, the occurrence of a CD24^hi^CD38^hi^ or CD24^hi^CD27^+^ phenotype for B cells does not necessarily reflect their functionality. In fact, in patients with autoimmune diseases, CD24^hi^CD38^hi^ and CD24^hi^CD27^+^ B cells hardly process immunosuppressive functions directly^8,9^. Direct evidence supporting a role for functional B_reg_ cells in the immunopathogenesis of human diseases is still lacking. Furthermore, a related issue that must be addressed in this context is whether functional B_reg_ cells exhibit unique functions that are different from those functions that are already known and, if so, how B cells exert these effects.

Metabolic reprogramming is emerging as a crucial process for reshaping immune cell differentiation and function^10,11^. Oxidative phosphorylation (OXPHOS) is employed by naïve and memory B cells to supply the basal energy needs of metabolic quiescence^12^. In contrast, high-strength glycolysis together with aggressive OXPHOS controls the antibody secretion of effector B cells (also termed plasma cells), and this process depends on active glucose and amino acid uptake machinery^13,14^. Notably, although not directly related to B cells, glycolysis is also considered an efficient activator of the inflammatory response^15,16^, whereas OXPHOS has been implicated in the anti-inflammatory response and immune tolerance^17,18^. To date, the precise metabolic programs of IL-10-secreting functional B_reg_ cells under pathological conditions are not known. In other words, evaluating the metabolic programs of functional B_reg_ cells in pathological conditions is crucial for understanding the polarization and functional status of these cells in immunopathogenesis.

Systemic lupus erythematosus (SLE) is a potentially fatal autoimmune disease characterized by extensive inflammation and tissue damage that can affect any part of the body. In patients with SLE, defects in suppressive cell functions are necessary for enhanced inflammation and the active stage of disease^19,20^. It has been assumed that B_reg_ cells are a protective factor that impedes the inflammatory response based on their ability to produce IL-10^1–4^. However, by probing the phenotypic features and functions of IL-10-secreting B cells in patients with SLE, we herein report that functional B_reg_ cells mainly serve as a pathogenic factor but do not serve as a suppressor of inflammation. Functional B_reg_ cells triggered by SLE pathological factors (e.g., CPG-DNA) display a previously unrecognized CD24^int^CD27^−^CD38^−^CD69^+/hi^ phenotype and simultaneously produce high amounts of inflammatory mediators, and this process is dominated by MAPK/ERK/P38 signal-elicited sequential oncogenic c-Myc upregulation and enhanced glycolysis. More importantly, we demonstrate that these inflammatory features repurpose B_reg_ cell functions away from those inducing CD8^+^ T cell tolerance and toward those inducing an inflammatory T_H_ cell response. Accordingly, inhibiting the activity of ERK, P38, c-Myc, or the glycolytic enzyme PFKFB3 in functional B_reg_ cells from patients with SLE effectively impairs the polarization of pathogenic inflammatory T_H_ cell subsets.

## Results

### Polarization of unrecognized functional B_reg_ cells in human SLE correlates with disease progression

**Fig. 1:**
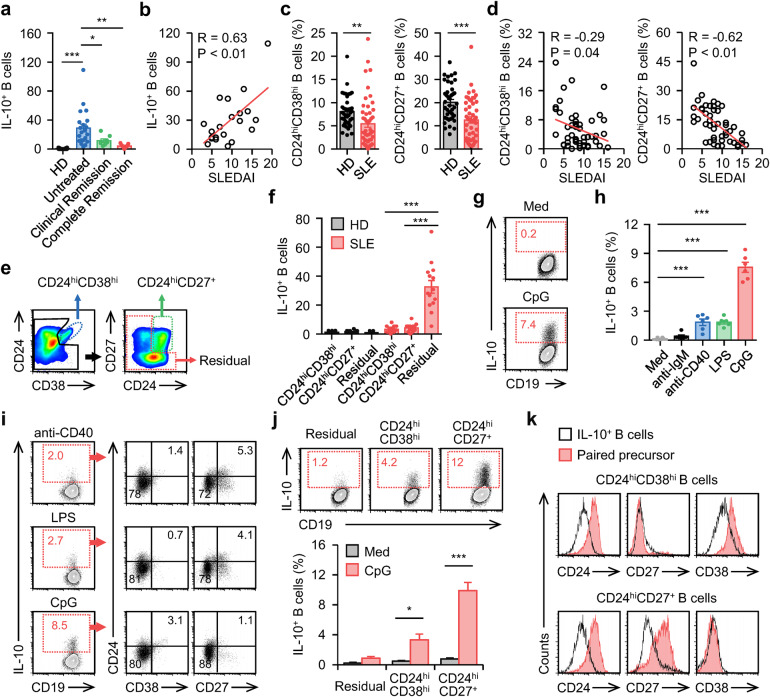
Polarization of IL-10-producing B_reg_ cells in human SLE **a** Enzyme-linked immunospot (ELISpot) detection of IL-10 production by circulating B cells from 20 healthy donors (HD), 21 untreated SLE patients, 8 clinical remission SLE patients, and 7 complete remission SLE patients. **b** Associations of circulating IL-10^+^ B cells with patients’ SLE disease activity index (SLEDAI) (*n* = 21). **c** FACS analysis of CD24^hi^CD38^hi^ and CD24^hi^CD27^+^ B cells from healthy donors (*n* = 40) and untreated SLE patients. **d** Associations of circulating CD24^hi^CD38^hi^ and CD24^hi^CD27^+^ B cells with patients’ SLEDAI (*n* = 49). **e** CD24^hi^CD38^hi^, CD24^hi^CD27^+^, and residual B cells from HD and untreated SLE patients were sorted by FACS. **f** IL-10 production by these cells were detected using ELISpot (*n* = 14). **g**–**i** Total B cells from HD were cultured in medium or treated with an anti-IgM antibody, anti-CD40 antibody, LPS (**h**, **i**), or CPG-DNA ODN (CPG) (**g**–**i**) for 3 d. IL-10^+^ (**g**, **h**), CD24^hi^CD38^hi^ (**i**), and CD24^hi^CD27^+^ (**i**) B cells were detected by FACS (*n* = 6). **j**, **k** CD24^hi^CD38^hi^, CD24^hi^CD27^+^, and residual B cells from HD were cultured in medium or were treated with CPG-DNA for 3 d. IL-10^+^ (**j**, **k**), CD24^hi^CD38^hi^ (**k**), and CD24^hi^CD27^+^ (**k**) B cells were detected by FACS (*n* = 5). Data were acquired from more than four independent experiments and shown as means ± SEM. Pearson’s correlation analysis for **b** and **d**. Significance was examined with one-way ANOVA test (**a**, **f**, **h**) or Student’s *t* test (**c**, **j**), *p < 0.05, **p < 0.01, ***p < 0.001

We used an ELISpot detection system to analyze IL-10 production by B cells purified from blood samples of 20 healthy donors, 21 untreated SLE patients, 8 SLE patients with clinical remission, and 7 SLE patients with complete remission (Supplementary Table 1). IL-10 was hardly secreted by B cells from the blood of healthy donors (Fig. 1a). Unexpectedly, this cytokine was spontaneously produced by a fraction of B cells from the blood of untreated SLE patients (Fig. 1a), and the density of these IL-10^+^ B cells positively correlated with disease progression (Fig. 1b and Supplementary Fig. 1a) but declined in the blood of SLE patients with clinical remission or complete remission (Fig. 1a). Thus, IL-10^+^ functional B_reg_ cells accumulated in the blood of SLE patients with active disease. CD24^hi^CD38^hi^ and CD24^hi^CD27^+^ B cells are considered the major cellular sources of peripheral B_reg_ cells in humans^8,9^. However, inconsistent with the above observations, the proportions of CD24^hi^CD38^hi^ and CD24^hi^CD27^+^ B cells in the blood of SLE patients were profoundly decreased (Fig. 1c and Supplementary Fig. 1b), and these decreases were also positively correlated with active disease (Fig. 1d and Supplementary Fig. 1c), suggesting that functional B_reg_ cells exhibit a phenotype unrelated to the CD24^hi^CD38^hi^ or CD24^hi^CD27^+^ phenotype. To test this hypothesis, we sorted peripheral CD24^hi^CD38^hi^ and CD24^hi^CD27^+^ B cells from healthy donors and SLE patients (Fig. 1e and Supplementary Fig. 1d) and probed their ability to spontaneously secrete IL-10. In all FACS-sorted CD24^hi^CD38^hi^ and CD24^hi^CD27^+^ B cells, IL-10 signals were rarely detected (Fig. 1f). By comparison, significantly higher frequencies of IL-10 signals were observed in residual B cells from SLE patients (Fig. 1f). This prompted us to further investigate the environmental factors and cellular sources that contribute to the polarization of functional B_reg_ cells in SLE patients.

It is noteworthy that elevated levels of plasma DNA enriched in hypomethylated CPGs are a biomarker of active SLE^21^. Therefore, we determined whether CPG-DNA promotes the polarization of functional B_reg_ cells. As shown in Fig. 1g, exposing total B cells from healthy donors to CPG-DNA for 3 days led to marked polarization of functional B_reg_ cells. Other environmental factors, including anti-IgM [B cell receptor (BCR)-triggering] antibodies, anti-CD40 [a B cell costimulatory signal], and LPS (a TLR4 agonist)^22–24^, also triggered polarization of IL-10^+^ functional B_reg_ cells, although to a lesser extent (Fig. 1h). In support of in vivo observations (Fig. 1f), the functional B_reg_ cells generated in vitro mainly exhibited a unique CD38^−^CD24^int^CD27^−^ phenotype (Fig. 1i). Furthermore, we also assessed the influences of CPG-DNA on FACS-sorted CD24^hi^CD38^hi^ and CD24^hi^CD27^+^ B cells, as well as residual B cells. Unexpectedly, CD24^hi^CD38^hi^ and CD24^hi^CD27^+^ B cells, but not the residual B cells, represented major cell populations that could be differentiated into functional B_reg_ cells (Fig. 1j). Notably, the functional B_reg_ cells polarized from CD24^hi^CD38^hi^ or CD24^hi^CD27^+^ B cells displayed a phenotype unrelated to CD24^hi^, CD27^+^, and/or CD38^hi^ (Fig. 1k), which is consistent with the finding that, in the blood of SLE patients, increasing frequencies of functional B_reg_ cells were accompanied by decreasing ratios of CD24^hi^CD38^hi^ and CD24^hi^CD27^+^ B cells (Fig. 1a, c).

### Functional B_reg_ cells exhibit a unique CD25^+^CD69^+/hi^CD80^+^CD86^+/hi^ phenotype that differs from that of their precursors

**Fig. 2:**
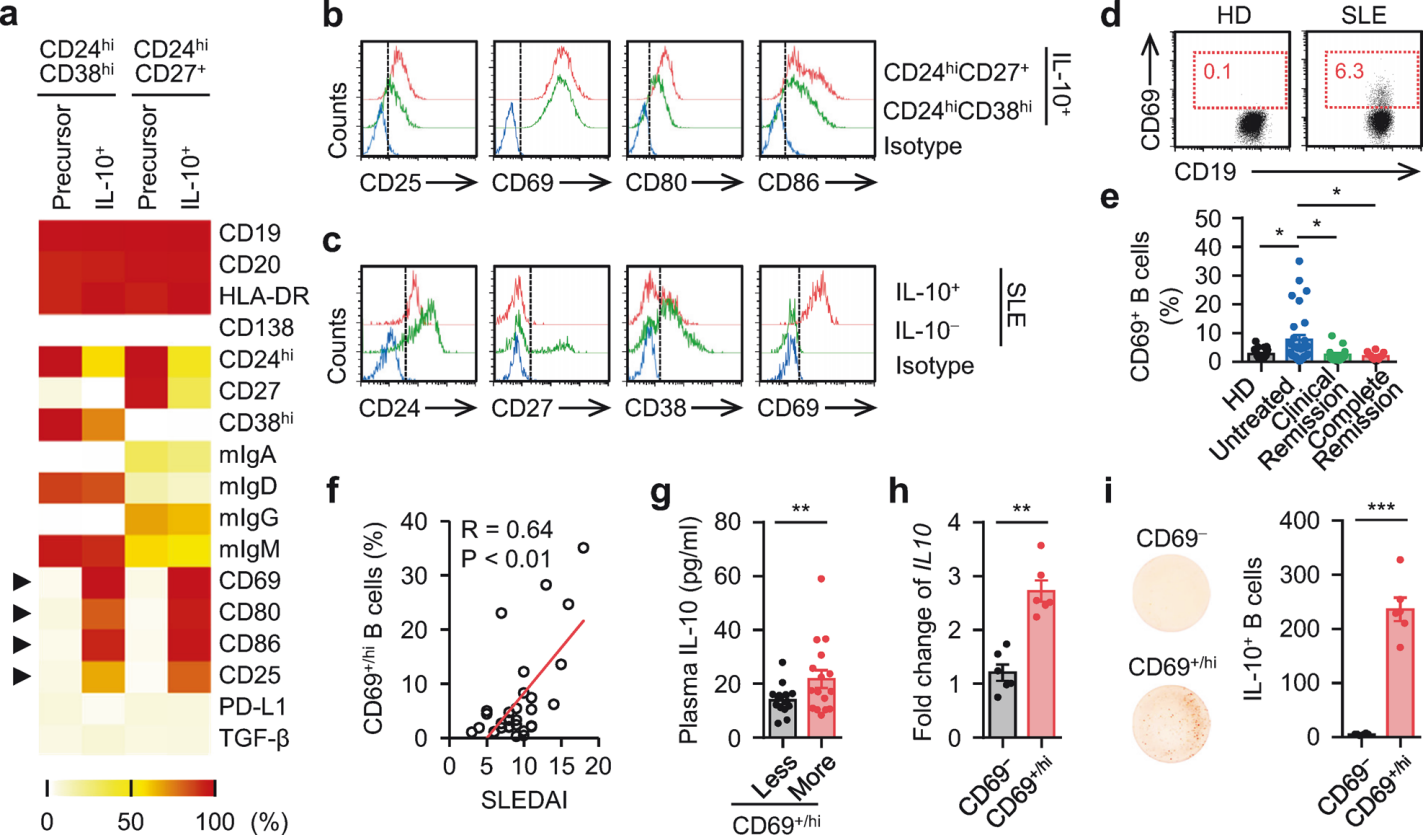
Phenotypic characteristics of IL-10-secreting B_reg_ cells in SLE **a**, **b** FACS analysis of the phenotypic characteristics of the untreated CD24^hi^CD38^hi^ and CD24^hi^CD27^+^ B cells (precursor), as well as IL-10^+^ cells derived from CD24^hi^CD38^hi^ and CD24^hi^CD27^+^ B cells by CPG-DNA induction (**a**). Representative histogram of CD25, CD69, CD80, and CD86 expression were shown in **b** (*n* = 5). **c** Expression of CD24, CD27, CD38, and CD69 in IL-10^+^ B cells purified from untreated SLE patients was detected by FACS (*n* = 6). **d**, **e** FACS analysis of CD69^+/hi^ B cells from healthy donors and untreated SLE patients. Representative FACS plots were shown in **d**. The portions of CD69^+/hi^ B cells relative to total B cells from 21 healthy donors, 30 untreated SLE patents, 14 clinical remission SLE patients, and 12 complete remission SLE patients were shown in **e**. **f**, **g** Associations of circulating CD69^+/hi^ B cells with disease progression (**f**) and plasma level of IL-10 from untreated SLE patients (**g**) (*n* = 30). **h**, **i** Expression of IL-10 by CD69^−^ and CD69^+/hi^ B cells isolated from untreated SLE patients were detected by PCR (**h**) and ELISpot (**i**), respectively (each *n* = 6). Data were acquired from more than four independent experiments and shown as means ± SEM. Pearson’s correlation analysis for **f**. Significance was determined with one-way ANOVA test (**e**), Student’s *t* test (**g**–**i**). *p < 0.05, **p < 0.01, ***p < 0.001

We next investigated the phenotypic characteristics of functional B_reg_ cells. Generally, CPG-DNA-induced B_reg_ cells, as well as their precursors CD24^hi^CD38^hi^ and CD24^hi^CD27^+^ B cells, displayed a non-plasma cell phenotype with substantial CD19, CD20, and HLA-DR but rare CD138 expression (Fig. 2a). Compared with CD24^hi^CD38^hi^ B cells, CD24^hi^CD27^+^ B cells displaying a memory phenotype and expressed higher frequencies of membrane immunoglobulin A (mIgA) and mIgG but lower levels of mIgD and mIgM. Interestingly, unlike the expression patterns of CD24, CD27, and CD38, the expression profiles of mIg in CD24^hi^CD38^hi^ and CD24^hi^CD27^+^ B cells remained virtually unchanged after their differentiation into functional B_reg_ cells (Fig. 2a), indicating that the generation of functional B_reg_ cells is a process unrelated to Ig class-switch recombination. Notably, although CPG-DNA-induced B_reg_ cells lost the conventional phenotypic features related to their precursors, these cells developed a unique CD25^+^CD69^+/hi^CD80^+^CD86^+^ phenotype (Fig. 2a, b). These data suggest that functional B cells purified from SLE patients directly may exhibit a phenotype that is related to CD24^int^CD27^−^CD38^−^CD69^+/hi^. In support, IL-10^+^ B cells from blood of SLE patients did displayed a CD24^dim/−^CD27^lo/−^CD38^lo/−^CD69^+/hi^ phenotype (Fig. 2c and Supplementary Fig. 2a).

Based on the above-mentioned findings, we proposed that CD69^+/hi^ B cells were more potent in producing IL-10 functionally. In fact, significantly higher frequencies of CD69^+/hi^ B cells were detected in the blood of untreated SLE patients than in blood from healthy donors (Fig. 2d, e), and the proportion of these cells positively correlated with disease progression in patients (Fig. 2f and Supplementary Fig. 2b). Of note, increased levels of plasma IL-10 in patients with increased frequencies of peripheral CD69^+/hi^ B cells were detected (Fig. 2g) and most CD69^+/hi^ B cells in blood of SLE patients were CD24^lo/−^CD27^lo/−^CD38^lo/−^ (Supplementary Fig. 2c). We subsequently sorted the CD69^+/hi^ B cells from the blood of SLE patients. In agreement with our hypothesis, CD69^+/hi^ B cells had more potential to express IL-10 mRNA (Fig. 2h). Indeed, the CD69^+/hi^ B cells displayed a 50-fold increase in IL-10 production compared with the CD69^−^ population (Fig. 2i).

### Functional B_reg_ cells co-polarize with inflammatory B cell populations

**Fig. 3:**
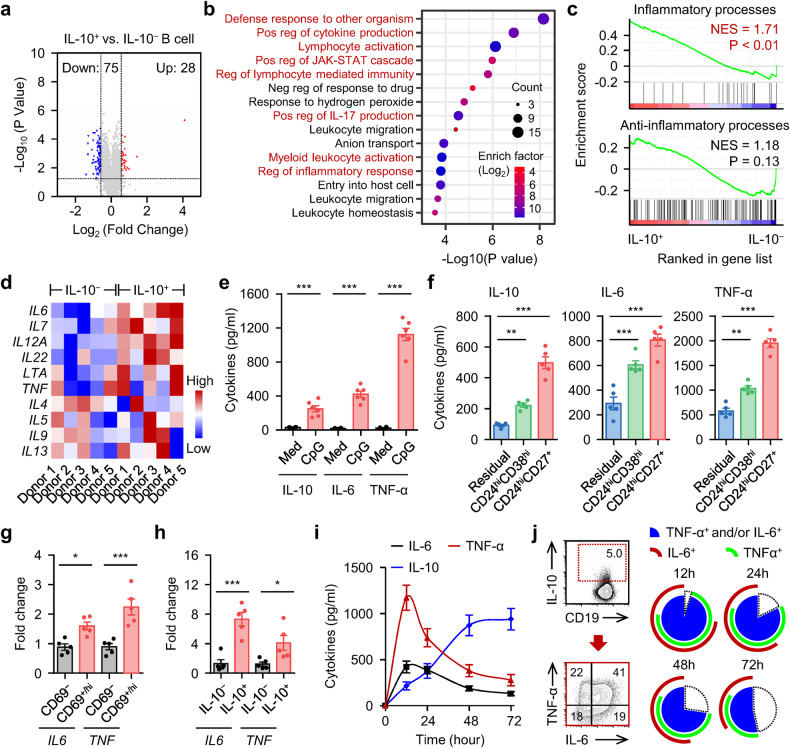
Inflammatory features of IL-10-secreting B_reg_ cells in SLE **a** The changes of genes in IL-10^+^ B cells versus IL-10^−^ B cells (GSE50895) were shown in volcano plot. The *P* value of each gene was calculated using DESeq2 and adjusted with Benjamini-Hochberg false discovery rate correction (*n* = 5). **b** Functional annotation of 103 differential gene expression listed in **a** were analyzed by DAVID tool. The top 15 enrichment GO terms are listed. **c** GSEA of inflammatory processes (WP530) and anti-inflammatory processes (GO: 0050728) in IL-10^+^ B cells versus IL-10^−^ B cells. NES, normalized enrichment score. **d** Heat map displaying inflammatory and anti-inflammatory cytokine expression in IL-10^+^ B cells versus IL-10^−^ B cells (*n* = 5). **e**, **f** Purified total B cells (**e**), CD24^hi^CD38^hi^, CD24^hi^CD27^+^, and residual B cells (**f**) from HD were cultured in medium or were treated with CPG-DNA for 12 h. IL-10, IL-6, and TNF-α production were detected by ELISA (*n* = 6). **g**, **h** Expression of *IL6* and *TNF* in CD69^−^ and CD69^+/hi^ B cells (**g**), or in IL-10^−^ and IL-10^+^ B cells (**h**) isolated from untreated SLE patients were detected by real-time PCR (*n* = 5). **i**, **j** Total B cells purified from HD were cultured in medium or treated with CPG-DNA. Dynamics of IL-10, IL-6, and TNF-α production was measured over the indicated times by ELISA (**i**). Frequencies of TNF-α^+^ and/or IL-6^+^ and TNF-α^−^IL-6^−^ B cells relative to total B-cell were examined by FACS (**j**) (*n* = 6 for **i**; *n* = 5 for **j**). Data were acquired from more than four independent experiments and shown as means ± SEM. Significance was determined with Student’s *t-*test (**e**, **g**, **h**) or one-way ANOVA test (**f**). *p < 0.05, **p < 0.01, ***p < 0.001

To further probe the characteristics of functional B_reg_ cells, we analyzed the transcriptional profiles of those cells with RNA sequencing (GSE50895). We identified 103 genes upregulated or downregulated at least 1.5-fold in functional B_reg_ cells (IL-10^+^ B cells) and annotated these genes using Gene Ontology (GO) (Fig. 3a, b). Surprisingly, although IL-10 itself is classically considered anti-inflammatory, activation of pathways related to the anti-inflammatory response was completely lacking (Fig. 3b). Instead, pathways related to inflammatory processes, including cytokine production, the defense response against organisms, and the activation of lymphocytes or myeloid cells, were intensively enriched (Fig. 3b), which was further confirmed by Gene Set Enrichment Analysis (GSEA) (Fig. 3c and Supplementary Fig. 3a, b). In support of these findings, significantly higher *IL6*, *IL7*, *IL12A*, *IL22*, *LTA*, and *TNF* levels were detected in IL-10^+^ B cells than in their IL-10^−^ counterparts (Fig. 3d). In contrast, irregular variations in the levels of the anti-inflammatory molecules *IL4*, *IL5*, *IL9*, or *IL13* were detected (Fig. 3d).

To ascertain whether functional B_reg_ cells and inflamed B cells are interrelated, we tested the inflammatory profiles of functional B_reg_ cells generated in vitro. Reliably, exposure to CPG-DNA, a factor contributing to functional B_reg_ cell generation, also resulted in substantial production of TNF-α and IL-6 by these cells (Fig. 3e). Analogously, CD24^hi^CD38^hi^ and CD24^hi^CD27^+^ B cells, two major groups of functional B_reg_ cell precursors, secreted significantly more IL-6 and TNF-α than other B cell populations (Fig. 3f). In parallel, CD69^+/hi^ B cells and IL-10^+^ B cells, both directly isolated from the blood of SLE patients, expressed higher levels of *IL6* and *TNF* than other B cell populations (Fig. 3g, h). Furthermore, measurement of the cytokines produced by B cells exposed to CPG-DNA over time revealed rapid accumulation of TNF-α and IL-6 in culture supernatants, with a maximum or a plateau reached within 24 h, followed by a gradual decline (Fig. 3i). In contrast, accumulation of IL-10 was delayed, but a sustained elevation did last for at least 3 days after stimulation (Fig. 3i). Given these results, we concluded that IL-10^+^ B cells were IL-6^+^ and/or TNF-α^+^ at the early measurement time point, and these cells, at the subsequent measurement time points, gradually and partially lost the ability to secrete these cytokines (Fig. 3j).

### Metabolic program that acts in differentiation of functional B_reg_ cells in SLE patients

**Fig. 4:**
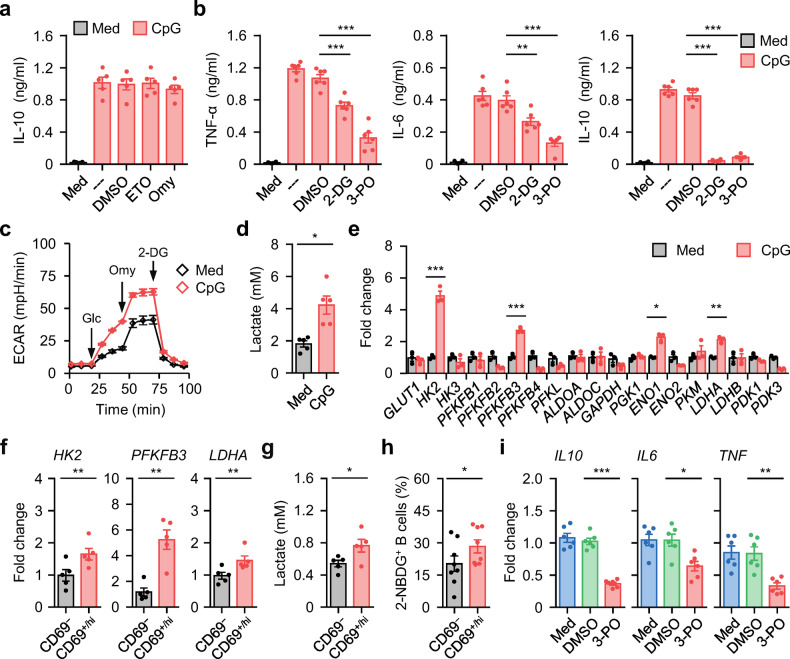
Metabolic reprogramming involved in inflammatory B_reg_ cell induction **a**, **b** Total B cells from healthy donors were cultured in medium or pretreated with DMSO, inhibitors against fatty acid oxidation (Etomoxir, ETO) (**a**), oxidative phosphorylation (Oligomycin, Omy) (**a**), or glycolysis (2-DG; 3-PO) (**b**). Thereafter, the cells were incubated in the presence or absence of CPG-DNA. Cytokine production were detected by ELISA (*n* = 5 for **a**; *n* = 6 for **b**). **c**–**e** Total B cells purified from healthy donors were cultured in medium or treated with CPG-DNA for 3 d. Extracellular acidification (ECAR) (**c**), accumulated lactate production (**d**), and expression of glycolytic enzymes (**e**) were measured by seahorse analyzer, lactate assay kit, and real-time PCR, respectively (*n* = 3 for **c** and **e**; *n* = 5 for **d**). **f**–**h** Expression of key rate-limiting glycolytic enzymes (**f**), accumulated lactate production (**g**), and capabilities of glucose incorporation (2-NBDG^+^) (**h**) by CD69^−^ and CD69^+/hi^ B cells purified from untreated SLE patients were examined by real-time PCR (**f**), lactate assay kit (**g**), and FACS (**h**), respectively (*n* = 5 for **f** and **g**; *n* = 8 for **h**). **i** Purified CD69^+/hi^ B cells from blood of untreated SLE patients were cultured in medium or in the presence of DMSO, or 3-PO for 12 h. Expression of *IL10*, *IL6*, and *TNF* were examined by real-time PCR. Data were acquired from more than four independent experiments and shown as means ± SEM. Significance was determined with one-way ANOVA test (**a**, **b**, **i**) or Student’s *t* test (**d**–**h**). *p < 0.05, **p < 0.01, ***p < 0.001

Metabolic reprogramming is essential for both lineage differentiation and the function of immune cells^10,11^. Notably, regulatory T (T_reg_) cells depend on OXPHOS and fatty acid metabolism^11,17^. To investigate whether such a metabolic program is also employed by functional B_reg_ cells, we exposed CPG-DNA-treated B cells to etomoxir (ETO), a widely used small-molecule inhibitor of fatty acid oxidation^25^, or to oligomycin (Omy), an inhibitor of ATP synthase;^26^ however, we detected negligible changes in CPG-DNA-elicited functional B_reg_ cell polarization by measuring the IL-10 concentration (Fig. 4a and Supplementary Fig. 4a, b), suggesting that OXPHOS and fatty acid metabolism are not involved.

Considering the requirement of glycolysis during the inflammatory response, CPG-DNA-treated B cells were incubated with 2-deoxyglucose (2-DG), a glucose analog that inhibits glucose incorporation^27^, or with 3-(3-pyridinyl)−1-(4-pyridinyl)−2-propen-1-one (3-PO), an inhibitor of the glycolytic enzyme PFKFB3^28^. Interestingly, cells that received either treatment not only exhibited decreased production of the inflammatory molecules TNF-α and IL-6 but also completely lost their ability to polarize into functional B_reg_ cells (Fig. 4b and Supplementary Fig. 4a, b). Thus, increased glycolysis may represent a cardinal feature during functional B_reg_ cell polarization. Consistent with this finding, after B cells were exposed to CPG-DNA, there was a rapid increase in the extracellular acidification rate (ECAR) (Fig. 4c and Supplementary Fig. 4c) and the extracellular lactate concentration (Fig. 4d). We further established that B cells undergoing CPG-DNA stimulation expressed significantly increased expression of the glycolytic enzymes *HK2*, *PFKFB3*, and *LDHA* (Fig. 4e). In line with this, CD69^+/hi^ B cells, as well as IL-10^+^ B cells, purified from SLE patients showed a similar expression pattern of glycolytic enzymes (Fig. 4f and Supplementary Fig. 4d), and these cells cultured ex vivo produced significantly more lactate (Fig. 4g) and displayed a greater capacity to incorporate the fluorescent glucose analog 2-(N-[7-nitrobenz-2-oxa-1,3-diazol-4-yl] amino)−2-deoxyglucose (2-NBDG) (Fig. 4h and Supplementary Fig. 4e). Supporting our hypothesis, suppression of glycolysis in CD69^+/hi^ B cells ex vivo by 3-PO successfully impeded their B_reg_ cell signature and inflammatory features (Fig. 4i).

### MAPK-mediated c-Myc signaling is essential for glycolysis-triggered functional B_reg_ cells

**Fig. 5:**
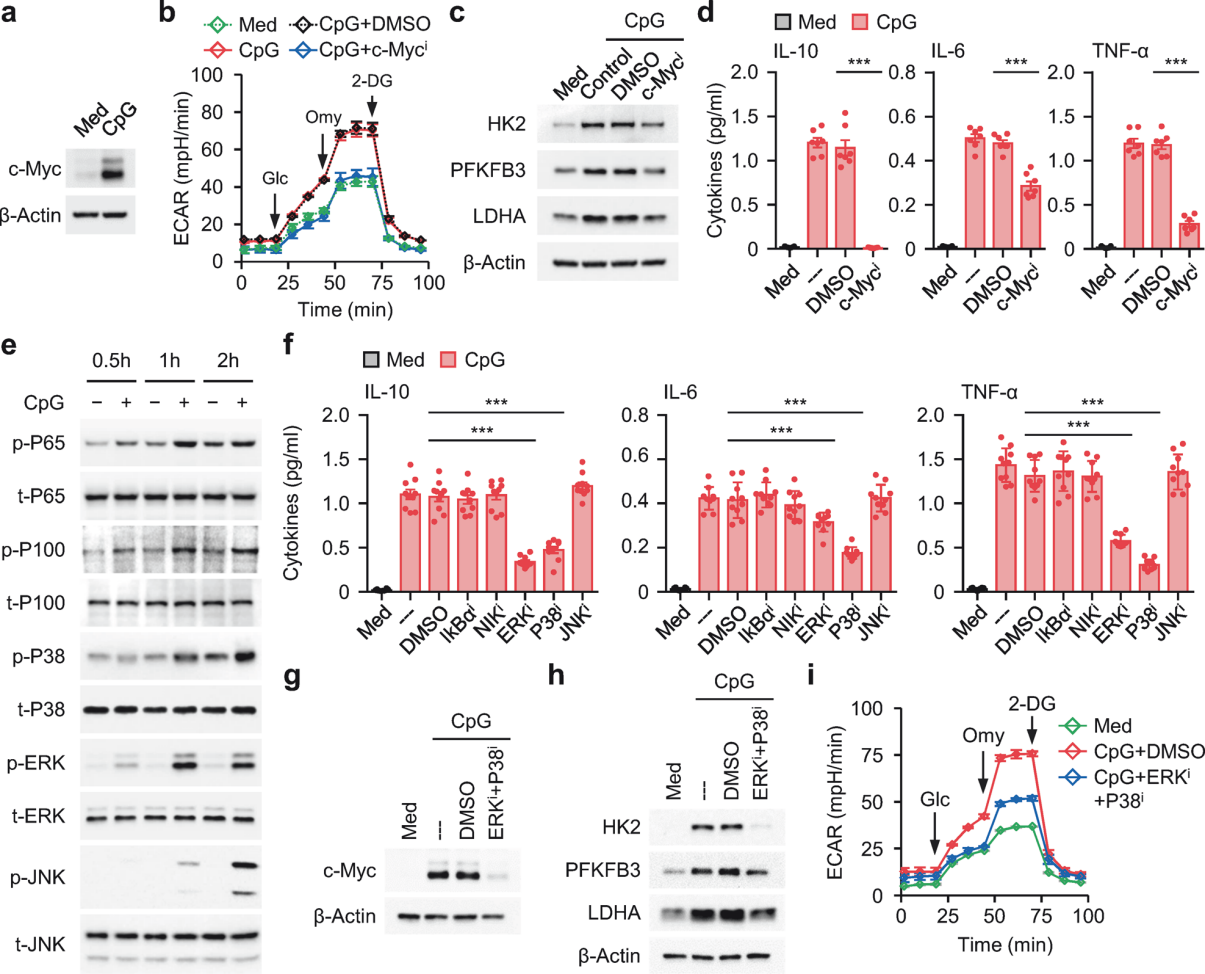
Signals and transcription factors that triggered B-cell glycolysis and inflammatory B_reg_ cell differentiation **a** Total B cells purified from healthy donors were cultured in medium or treated with CPG-DNA. c-Myc expression was examined by immunoblotting (*n* = 3). **b**–**d** Total B cells purified from healthy donors were cultured in medium or pretreated with DMSO, or inhibitor against c-Myc signals. Thereafter, the cells were cultured in the presence or absence of CPG-DNA. Basal extracellular acidification (**b**), expression of glycolytic enzymes (**c**), and cytokine secretion (**d**) were detected by seahorse analyzer, immunoblotting, and ELISA, respectively (*n* = 3 for **b** and **c**; *n* = 7 for **d**). **e** Purified total B cells from healthy donors were cultured in medium or stimulated with CPG-DNA. Activation of MAP kinase and NF-κB signals were determined by immunoblotting (*n* = 3). **f**–**i** Total B cells purified from healthy donors were cultured in medium or pretreated with DMSO, or inhibitor against MAP kinase (**f**–**i**) or NF-κB signals (**f**). Thereafter, the cells were cultured in the presence or absence of CPG-DNA. Cytokine production (**f**), expression of c-Myc (**g**) and glycolytic enzymes (**h**), and extracellular acidification (**i**) were detected by immunoblotting, seahorse analyzer, and ELISA, respectively (*n* = 10 for **f**; *n* = 3 for **g**–**i**). Data were acquired from more than four independent experiments and shown as means ± SEM. Significance was determined with one-way ANOVA test (**d**, **f**). ***p < 0.001

It is generally thought that increased AKT/mTOR signaling induces HIF-1α activity and subsequent glycolysis^29^. Indeed, exposing B cells to CPG-DNA triggered marked AKT/mTOR activation and HIF-1α upregulation (Supplementary Fig. 5a), which suggested the involvement of the AKT-mTOR-HIF-1α pathway during glycolysis-elicited functional B_reg_ cell polarization. However, this assumption was rapidly refuted by further observation that inhibiting the activity of either mTOR or HIF-1α in CPG-DNA-treated B cells did not affect the increase in the ECAR (Supplementary Fig. 5b), the upregulation of the glycolytic enzymes *HK2*, *PFKFB3*, and *LDHA* (Supplementary Fig. 5c, d), the production of the inflammatory mediators IL-6 and TNF-α, or the polarization of functional B_reg_ cells (Supplementary Fig. 5e). Notably, it is also reported that c-myc can regulate energy metabolism by directly activating genes involved in glycolysis, glutamine metabolism and mitochondrial biogenesis^30^. Interestingly, via GSEA, we noted that the c-Myc signaling pathway was intensively enriched in IL-10^+^ B cells (Supplementary Fig. 5f, g). Additionally, c-Myc was robustly upregulated in B cells exposed to CPG-DNA (Fig. 5a). In agreement with these findings, suppressing c-Myc signaling in either CPG-DNA-treated B cells or IL-10^+^ B cells purified from blood of SLE patients efficiently decreased glycolysis (Fig. 5b, c and Supplementary Fig. 5h), as well as the subsequent inflammatory response and functional B_reg_ cell generation (Fig. 5d and Supplementary Fig. 5i).

To further explore the mechanisms involved in triggering functional B_reg_ cells, we analyzed the activation kinetics of the MAPK and canonical and noncanonical NFκB pathways. After B cells were exposed to CPG-DNA, all the signaling pathways analyzed were rapidly activated, although to varying extents (Fig. 5e). Notably, using inhibitors to block the signal transduction of ERK and P38 in B cells effectively impaired the CPG-DNA-triggered inflammatory response and functional B_reg_ cell generation, whereas abolishing the phosphorylation of JNK or the NF-κB subunits P65 and P100 had only a marginal effect (Fig. 5f). In support of the above-mentioned findings that c-Myc-mediated glycolysis was a key upstream event for functional B_reg_ cell generation, we established that combined inhibition of ERK and P38 in B cells effectively abrogated CPG-DNA-mediated c-Myc upregulation (Fig. 5g) and subsequent glycolysis (Fig. 5h, i).

### Targeting c-Myc-elicited glycolysis abolishes the pathogenic response induced by functional B_reg_ cells

**Fig. 6:**
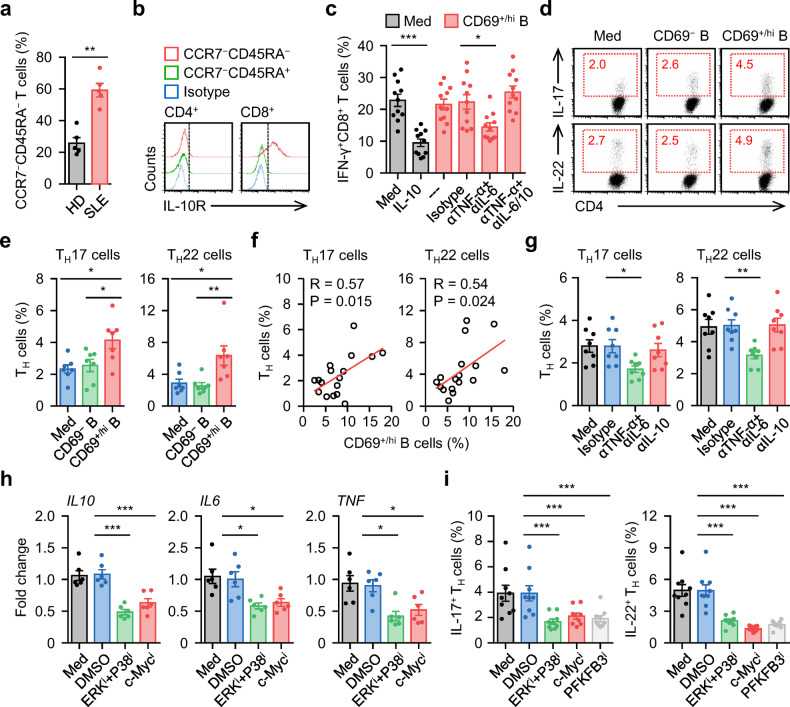
Targeting c-Myc-elicited glycolysis abrogates inflammatory B_reg_ cell mediated pathogenic response **a**, **b** FACS analysis of CCR7^−^CD45RA^−^ effector memory cells T cells in circulating T cells (**a**) and IL-10R expression (**b**) on effector memory T cells in blood samples from 5 healthy donors and 5 untreated SLE patients. **c** Purified T cells from SLE patients were cultured in medium, treated with recombinant human IL-10, or cocultured with CD69^+/hi^ B cells in the presence of isotype control, anti-TNF-α antibody plus anti-IL-6 antibody, or anti-TNF-α antibody plus anti-IL-6 antibody plus anti-IL-10 antibody for 7 d. IFN-γ expression in CD8^+^ T cells was examined using FACS (*n* = 11). **d**, **e** Purified T cells from SLE patients were left untreated or cultured with CD69^−^ B cells or CD69^+/hi^ B cells for 7 d. Differentiation of T_H_17 and T_H_22 was determined by FACS (*n* = 7). Representative dotplots and statistical data were shown in **d** and **e**, respectively. **f** Associations of circulating CD69^+/hi^ B cells with circulating T_H_17 and T_H_22 cells in untreated SLE patients (*n* = 17). **g** Purified T cells from untreated SLE patients were cultured with autologous CD69^+/hi^ B cells in the presence of isotype control, anti-TNF-α antibody plus anti-IL-6 antibody, or anti-IL-10 antibody for 7 d. Differentiation of T_H_17 and T_H_22 was determined by FACS (*n* = 8). **h**, **i** Purified CD69^+/hi^ B cells from SLE patients were cultured in medium or pretreated with DMSO, inhibitor against c-Myc, PFKFB3, or ERK plus P38. Thereafter, the cells were incubated with autologous T cells for 7 d. Cytokine expression in B cells (**h**) and differentiation of T_H_17 and T_H_22 (**i**) was measured by real-time PCR and FACS (*n* = 6 for **h**; *n* = 9 for **i**). Data were acquired from more than four independent experiments and shown as means ± SEM. Pearson’s correlation analysis for **f**, significance was determined with Student’s *t* test (**a**) or one-way ANOVA test (**c**, **e**, **g**–**i**). *p < 0.05, **p < 0.01, ***p < 0.001

Given that functional B_reg_ cells (IL-10^+^ B cells) were co-polarized with inflammatory B cells and potently correlated with the disease progression of SLE patients (Figs. 1–3), we determined whether inflammatory features repurposed B_reg_ cell function away from immune tolerance and toward a pathogenic response. In the blood of SLE patients, most T cells mainly exhibited a CCR7^−^CD45RA^−^ effector memory phenotype (Fig. 6a). Analyzing the IL-10 receptor (IL-10R) in T cells from SLE blood revealed that this receptor was primarily expressed by CD8^+^ but not CD4^+^ T cells (Fig. 6b), implying that IL-10 mainly affects CD8^+^ T cell function. Accordingly, exposure of CD8^+^ T cells to recombinant human (rh) IL-10 led to a marked reduction in IFN-γ in cells (Fig. 6c). However, coculture of CD8^+^ T cells and autologous CD69^+/hi^ B cells, both of which were purified from the blood of SLE patients, showed marginal effects (Fig. 6c). Interestingly, in such a coculture system, adding antibodies against IL-6 plus TNF-α effectively restored CD69^+/hi^ B cell-mediated CD8^+^ T cell tolerance, and this process could be reversed by additional neutralization of IL-10 (Fig. 6c).

Next, we further considered the effects of functional B_reg_ cells on CD4^+^ T cell function. We found that exposure of CD4^+^ T cells to autologous CD69^+/hi^ B cells resulted in marked differentiation of T_H_17 and T_H_22 cells ex vivo (Fig. 6d, e). In the blood of SLE patients, the proportion of CD69^+/hi^ B cells positively correlated with that of T_H_17 or T_H_22 cells (Fig. 6f). Consistent with our hypothesis, blocking the effect of IL-6 plus TNF-α on CD4^+^ T cells successfully suppressed the polarization of T_H_17 and T_H_22 cells, but neutralizing IL-10 had minimal effects (Fig. 6g). Because MAPK-mediated c-Myc upregulation is also required for glycolysis-triggered B cell inflammatory effects (Fig. 5), we pretreated CD69^+/hi^ B cells with inhibitors of ERK, P38, c-Myc, or the glycolytic enzyme PFKFB3 before culturing them with autologous CD4^+^ T cells. Consistently, all of the pretreatment groups showed an attenuated inflammatory response of CD69^+/hi^ B cells and impaired subsequent differentiation of T_H_17 or T_H_22 cells (Fig. 4i, and Fig. 6h, i). These data indicate that these inflammatory features repurposed B_reg_ cell function away from inducing CD8^+^ T cell tolerance and toward inducing a pathogenic CD4^+^ T cell response.

## Discussion

Previous studies have implied a suppressive role of B_reg_ cells solely based on the anti-inflammatory signature of IL-10^1^. In the present investigation, by using SLE as a model system, we identified a disruptive role of IL-10-secreting functional B_reg_ cells in maintaining the pathogenic disease response. We substantially demonstrated that functional B_reg_ cells generated in pathological environments of SLE exhibit inflammatory features rather than anti-inflammatory features, suggesting an unrecognized immune-editing mechanism by which B cells regulate the progression of inflammatory diseases.

Despite recent success in identifying several subpopulations with B_reg_ cell potential, little is known about the phenotype and regulation of functional B_reg_ cells in humans^1^. At present, the conclusion that B_reg_ cells hamper the progression of autoimmune diseases remains controversial^31,32^. It has been suggested that CD24^hi^CD38^hi^ and CD24^hi^CD27^+^ B cells comprise the major cellular groups of peripheral B_reg_ cells in humans^8,9^. In fact, neither of these subpopulations isolated from healthy individuals or patients with SLE functionally produce IL-10. Hence, the ratio of CD24^hi^CD38^hi^ or CD24^hi^CD27^+^ B cells cannot reflect the contribution of functional B_reg_ cells to the progression of SLE. More strikingly, although IL-10 itself is anti-inflammatory, IL-10^+^ functional B_reg_ cells increase significantly with the progression of SLE, suggesting a pathogenic role. Of note, the environmental factors (e.g., CPG-DNA) that trigger functional B_reg_ cells in SLE also effectively contribute to the progression of disease^21^. It is plausible that IL-10 is merely a marker for functional B_reg_ cells and cannot represent all the functions of those cells. This notion is supported by our observation that functional B_reg_ cells display a previously unrecognized CD24^int^CD27^−^CD38^−^CD69^+/hi^ phenotype different from that of their precursors, CD24^hi^CD38^hi^ and CD24^hi^CD27^+^ B cells, and these cells produce high amounts of inflammatory mediators in their early differentiation stage. Thus, B_reg_ cells are versatile in that they create inflammation and/or suppression according to their differentiation stage and exogenous signals. Consistently, several groups also reported that B_reg_ cells can serve as killer cells, as described for CD8^+^ T or NK cells^7^.

In both humans and mice, glycolysis participated in M1 macrophage polarization^15^, whereas OXPHOS is restricted to M2 macrophages^18^. Analogously, increased OXPHOS maintains the suppressive activity of T_reg_ cells;^17^ whereas potent glycolysis is responsible for the effector activity of cytotoxic T cells^33^. Unexpectedly, the metabolic program employed by functional B_reg_ cells differed from that used by T_reg_ cells. We demonstrated that glycolysis, but not OXPHOS, is vital for the polarization and function of B_reg_ cells in SLE patients, and this was proved by four sets of experiments. First, the environmental factors (e.g., CPG-DNA) contributing to the induction of functional B_reg_ cells also result in increased expression of the glycolytic enzymes *HK2*, *PFKFB3*, and *LDHA*, as well as marked production of extracellular lactate. Second, blocking glucose incorporation or suppressing the activity of the glycolytic enzyme PFKFB3 in CPG-DNA-treated B cells successfully impairs the differentiation of functional B_reg_ cells in vitro, whereas incubating B cells with inhibitors of OXPHOS has only marginal effects. Third, functional B_reg_ cells isolated from SLE patients express higher levels of glycolytic enzymes, and when cultured ex vivo, these cells produce significantly increased levels of lactate and display a greater capacity to incorporate glucose. Fourth, suppressing glycolysis in CD69^+/hi^ functional B_reg_ cells ex vivo with 3-PO successfully impedes their B_reg_ cell signature and inflammatory features. Therefore, activation of glycolysis in cells with B_reg_ cell potential may represent a novel route for triggering a pathogenic response in SLE patients. This hypothesis is compatible with several previous studies showing that the induction of glycolysis in macrophages and dendritic cells also enhances the pathogenic response in inflammatory diseases^15,34^.

AKT/mTOR signal-triggered HIF-1α activity is essential for myeloid cell glycolysis and subsequent inflammatory responses^29^. However, although this pathway is also active in B cells treated with CPG-DNA, inhibiting mTOR and HIF-1α activity cannot attenuate glycolysis and the subsequent differentiation of functional B_reg_ cells, suggesting the involvement of other signaling pathways. Notably, Myc proteins regulate cell growth and are oncogenic in many cancers, particularly B cell lymphoma^30^. In fact, c-Myc is also crucial for CPG-DNA-mediated glycolysis and the subsequent differentiation of functional B_reg_ cells. More importantly, suppressing c-Myc signaling in functional B_reg_ cells ex vivo efficiently reverses the pathogenic effects of functional B_reg_ cells. Additionally, abolishing the phosphorylation of ERK and P38 in B cells effectively impairs c-Myc-triggered glycolysis, the inflammatory response and functional B_reg_ cell generation. These findings agree with data reported by Wei and colleagues showing that evolutionarily conserved MAPK/ERK pathway triggers T cell response via c-Myc-elicited glycolysis^35^. Thus, a comprehensive study of the signaling network of functional B_reg_ cells will be beneficial for designing rational therapeutic strategies for autoimmune diseases^36,37^.

Binding of IL-10R by IL-10 should trigger dysfunction of effector T cells^38,39^. The elevated expression of the proinflammatory factors IL-6 and TNF-α in functional B_reg_ cells in patients with SLE abrogates the likelihood of engagement of this pathway. Coincidentally, inflammatory TNF-α has also been proven to reverse the suppressive function of T_reg_ cells by phosphorylating Foxp3^40,41^. Thus, it is not the suppressive cells per se but rather the inflammatory “context” that determines the ability of the suppressive cells^42,43^. Moreover, during the progression of autoimmune diseases, T_H_ cells regularly display pathogenic function by triggering and sustaining inflammation^44,45^. The current study provides important new insights into the previously unrecognized helper role of functional B_reg_ cells. After exposure to inflammatory mediators released by functional B_reg_ cells, T_H_ cells obtain the ability to polarize into proinflammatory T_H_ subsets and that contribute to chronic inflammation.

In our current study, all data are obtained based on clinical observations and ex vivo (or in vitro) experiments using human primary immune cells. It should be emphasized that some of our findings may not fully reflect the in vivo conditions of SLE patients. However, in mouse lupus model, the progenitors and their phenotypic characteristics of functional B_reg_ cells are different from those of human functional B_reg_ cells^46^. Therefore, establishing a suitable animal model which can reproduce the immune environment of human SLE may help us to better understand the potential mechanism in the development of the disease.

## Materials And methods

### Patients

A total of 317 peripheral blood samples from SLE patients who met the Systemic Lupus International Collaborating Clinics classification criteria were collected at the First Affiliated Hospital of Nanjing Medical University and the Third Affiliated Hospital of Sun Yat-sen University from May 2018 to January 2022. 71 untreated patients were used for analyzing the clinical relevance of peripheral B cell subsets (Supplementary Table 1, cohort 1 and Supplementary Table 2); Another 219 untreated patients were enrolled for the isolation of leukocytes for subsequent ex vivo experiments (Supplementary Table 1, cohort 2); 15 asymptomatic clinical remission patients who may get treatment with hydroxychloroquine, glucocorticoids, or tacrolimus and another 12 complete remission patients who achieved a serologically and clinically quiescent period lasting for at least 2 years were also used for the isolation of leukocytes (Supplementary Table 1, cohort 3 and 4). Pregnant women and patients with severe infection, other systemic diseases were excluded in the study. The control samples were obtained from age-matched healthy volunteers registered in the physical examination center of the Third Affiliated Hospital of Sun Yat-sen University, who would be excluded once with other diagnosed systemic diseases, oral ulcer, skin rash, a complaint of joint symptoms, fever, and positive family history of SLE or other possible symptoms of SLE. All samples were anonymously coded in accordance with local ethical guidelines (as stipulated by the Declaration of Helsinki). Written informed consent was obtained from the patients, and the protocol was approved by the Review Board of Sun Yat-sen University.

### Peripheral blood mononuclear cells (PBMC) isolation

PBMC were purified by density gradient centrifugation in Ficoll. Thereafter, the PBMC were washed and resuspended in culture medium (RPMI 1640 + 10% FBS). B cells and T cells were isolated from the PBMC by magnetic separation with MACS column (Miltenyi Biotec). Thereafter, CD24^hi^CD38^hi^ and CD24^hi^CD27^+^ B cells, as well as CD69^+/hi^ and CD69^−^ B cells were sorted by FACS (MoFlo, Beckman Coulter). IL-10^−^ and IL-10^+^ B cells were further purified using the IL-10 secretion assay-detection kits. These cells were used in subsequent experiments.

### Immunoblotting

B cells from in vitro culture system were harvested and washed with PBS. Protein lysis and immunoblotting were conducted as previously described^16^. The antibodies used are shown in Supplementary Table 3.

### Flow cytometry (FACS)

B cells and T cells purified from blood samples were incubated with fluorochrome-conjugated antibodies and subsequently examined by FACS. Under certain circumstances, B cells and T cells from blood samples, in vitro or ex vivo coculture experiments were treated with Leukocyte Activation Cocktail (BD Bioscience) for 5 h at 37 °C. Afterward, the cells stained with surface markers were fixed and permeabilized using IntraPrep reagent (Beckman Coulter), and finally stained with intracellular markers. Data were obtained using Gallios flow cytometer (Beckman Coulter). Antibodies used are shown in Supplementary Table 4.

### Enzyme-linked immunospot assay (ELISpot)

ELISpot assays were conducted with kits (BD Bioscience). For IL-10 detection, 1 × 10^5^ B cells were cultured for 24 h. The images were obtained using an ELISpot reader (CTL). Thereafter, the spot numbers were calculated.

### Enzyme-linked immunosorbent assay (ELISA)

Concentrations of TNF-α, IL-6 and IL-10 in the culture supernatants or in the plasma from SLE patients were detected using ELISA kits purchased from eBioscience. Antibodies used are listed in Supplementary Table 5.

### Extracellular acidification (ECAR) analyses

The ECAR of B cells was measured via XF-24 Extracellular Flux Analyzer (Seahorse Bioscience). In some experiments, B cells were left untreated or were pretreated with DMSO, or inhibitor against mTOR, HIF-1α, c-Myc, ERK or P38 signals. Thereafter, the cells were incubated in the presence or absence of CPG-DNA for 12 h. B cells were suspended in XF Base Medium Minimal DMEM supplemented with L-Glutamine (2 mM) and then placed on a cell culture microplate (5 × 10^5^ cells/well; XF-24, Seahorse Bioscience). During real-time measurement of the ECAR, Glucose (10 mM), oligomycin A (1 μM), and 2DG (50 mM) were added to the cells. The related reagents used are shown in Supplementary Table 5.

### Glucose uptake assay

B cells were purified from SLE patients. Thereafter, these cells were cultured for 1 h in PBS and then incubated with fluorescence-labeled 2-NBDG for 0.5 h at 37 °C. Finally, the cells were harvested and examined by FACS (Gallios).

### Lactate assay

Purified B cells from healthy blood were cultured in medium or treated with CPG-DNA for 3 d. Thereafter, the culture supernatants were collected. In some cases, CD69^+/hi^ and CD69^−^ B cells purified from SLE were culture for 3 days. Concentrations of the lactate were determined using L-lactate assay Kit (eton bioscience). L-lactate assay Kit used are shown in Supplementary Table 5.

### In vitro or ex vivo experiments for B cells

In vitro experiments, purified B cells (Miltenyi Biotec) from healthy donor were left untreated or were treated with an anti-IgM antibody (5 μg/ml), an anti-CD40 antibody (2 μg/ml), LPS (5 μg/ml), or CpG-DNA (2.5 μg/ml). In some in vitro experiments, before treatment with CPG-DNA, B cells were incubated with DMSO, 2-DG (10 mM), or a specific inhibitor against glycolytic enzyme PFKFB3 (3-PO, 10 μM), ATP synthase (Oligomycin, 0.2 µM), fatty acid oxidation (etomoxir, 50 µM), mTOR (Rapamycin, 10 µM), HIF-1α (Echinomycin, 1 ng/ml), c-Myc ((JQ1, 20 μM), JNK (SP 600125, 5 μM), ERK (U0126, 10 μM), P38 (SB 203580, 10 μM), canonical NFκB (BAY 11-7082, 5 μM) or non-canonical NF-κB (Amgen16, 1 μM) signals. Thereafter, IL-10 production was detected by indicated time. In some ex vivo experiments, CD69^+/hi^ and CD69^−^, or IL-10^+^ and IL-10^−^ B cells purified from untreated SLE patients’ blood were left untreated or treated with DMSO, or a specific inhibitor against c-Myc (JQ1), PFKFB3 (3-PO) for 12 h, then the expression of cytokine and glycolytic enzyme were measured. The inhibitors are shown in Supplementary Table 5.

### Ex vivo coculture system of T cells

Purified T cells were cultured in medium, or treated with human IL-10 (10 ng/ml), or were cultured with autologous CD69^−^ or CD69^+/hi^ B cells in the presence of anti-CD3 (2.5 µg/ml) as well as anti-CD28 (2.5 µg/ml) antibodies (eBioscience) for 2 days. Thereafter, the cells were incubated for 5 d with IL-2 (20 IU/ml) (eBioscience). In some cases, CD69^+/hi^ B cells were cultured in medium or pretreated with a specific inhibitor against PFKFB3 (3-PO), c-Myc (JQ1), ERK (U0126) or P38 (SB 203580). Thereafter, the B cells were washed and cocultured with autologous T cells. In other experiments, T cells were incubated with blocking antibodies against IL-10 (10 µg/ml), IL-6 (25 µg/ml), or TNF-α (25 µg/ml) (R&D Systems) and afterward cultured with autologous B cells. The neutralizing antibodies are shown in Supplementary Table 5.

### Real-time polymerase chain reaction (PCR)

Total RNA of B cells from blood of SLE patients or from ex vivo or in vitro culture system were extracted using Trizol reagent (Invitrogen). PCR was conducted as previously described^16^. The primers are shown in Supplementary Table 6. All data are shown in arbitrary units compared with the expression of 18 S rRNA.

### Statistical analysis

The gene expression profiling of human IL-10^−^ and IL-10^+^ B cells were downloaded from GEO database (GSE50895). Data are presented as means ± SEM. All statistical tests were performed as two-sided. We applied the Student t test to compare normally distributed data; and the non-parametric exact Wilcoxon signed-rank test was used to compare data not normally distributed. For multiple comparisons, an analysis of variance followed by Bonferroni’s correction was applied. R values were calculated based on the analysis of Pearson’s correlation. All statistical tests were analyzed using GraphPad Prism (v.6) software. The *p* values < 0.05 were considered statistically significant.

### Supplementary information

Supplementary materials

## Data Availability

All data are available in the main text or the supplementary materials.
