# Correction: c-Myc-driven glycolysis polarizes functional regulatory B cells that trigger pathogenic inflammatory responses

**DOI:** 10.1038/s41392-024-01783-7

**Published:** 2024-03-22

**Authors:** Xu-Yan Wang, Yuan Wei, Bo Hu, Yuan Liao, Xiaodong Wang, Wen-Hua Wan, Chun-Xiang Huang, Mahepali Mahabati, Zheng-Yu Liu, Jing-Rui Qu, Xiao-Dan Chen, Dong-Ping Chen, Dong-Ming Kuang, Xue-Hao Wang, Yun Chen

**Affiliations:** 1 MOE Key Laboratory of Gene Function and Regulation, Guangdong Province Key Laboratory of Pharmaceutical Functional Genes, School of Life Sciences, Sun Yat-sen University, Guangzhou, China; 2 Department of Laboratory Medicine, the Third Affiliated Hospital of Sun Yat-sen University, Guangzhou, China; 3 School of Pharmaceutical Sciences, Shenzhen University Health Science Center, Shenzhen, China; 4 Hepatobiliary Center, The First Affiliated Hospital of Nanjing Medical University, Key Laboratory of Liver Transplantation, Chinese Academy of Medical Sciences, NHC Key Laboratory of Living Donor Liver Transplantation (Nanjing Medical University), Nanjing, China; 5 Department of Immunology, Key Laboratory of Human Functional Genomics of Jiangsu Province, Nanjing Medical University, Nanjing, China; 6 Jiangsu Key Lab of Cancer Biomarkers, Prevention and Treatment, Collaborative Innovation Center for Cancer Personalized Medicine, Nanjing Medical University, Nanjing, China

**Keywords:** Inflammation, Lymphocytes

Correction to: *Signal Transduction and Targeted Therapy* 10.1038/s41392-022-00948-6, published online 18 April 2022

In the process of collating the raw data, the authors noticed an inadvertent error occurred in Supplementary Fig. 5d that needs to be corrected after online publication of the article 1.

During the preparation of Supplementary Fig. 5d, the representative western blot image of HK2 was repeatedly inserted as PFKFB3 blot by mistake. The correct data are provided as follows.
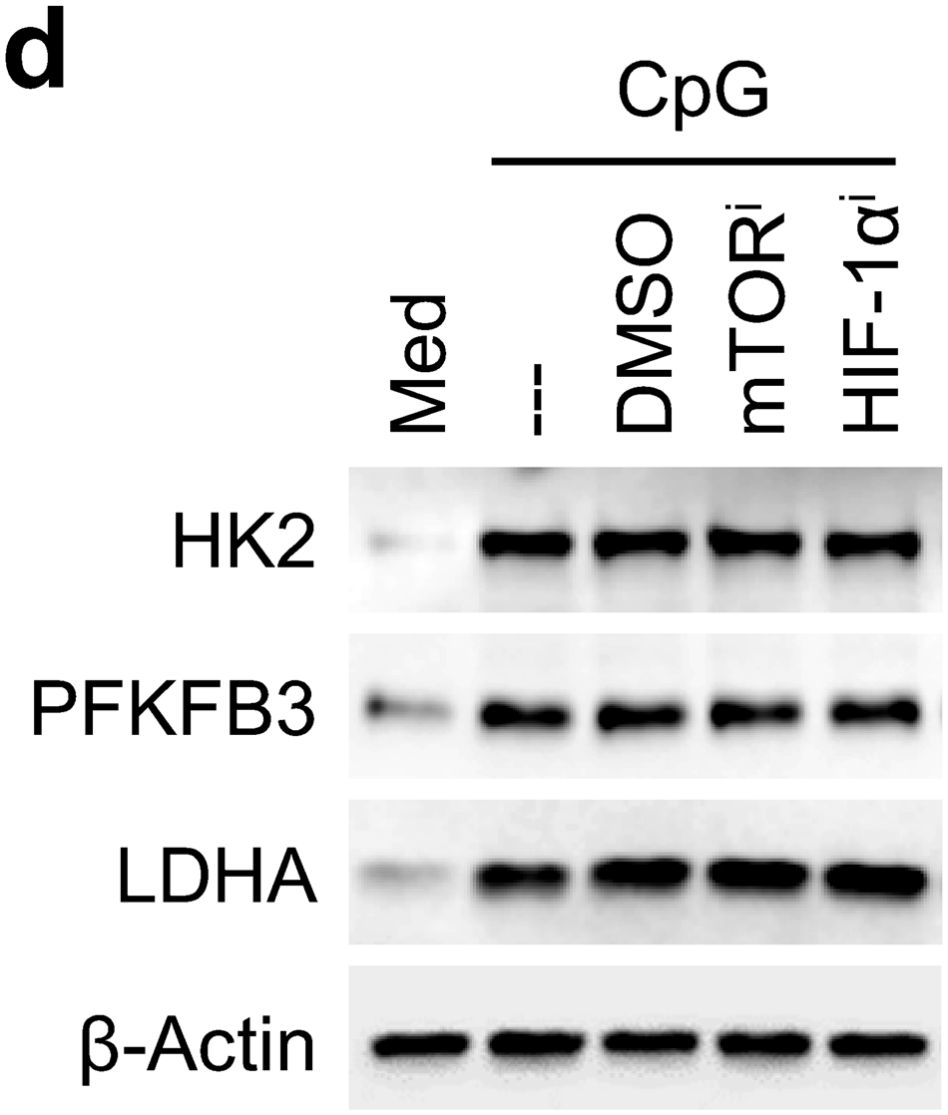


The key findings of the article are not affected by these corrections.

The original article has been corrected.

