# Supplementary material for: c-Myc-driven glycolysis polarizes functional regulatory B cells that trigger pathogenic inflammatory responses
Source: Signal Transduct Target Ther. 2022 Apr 18;7:105. doi: 10.1038/s41392-022-00948-6 (PMC9013717; doi:10.1038/s41392-022-00948-6)
Supplement: Supplementary file 1 — Supplementary materials [file 41392_2022_948_MOESM1_ESM.docx]

Supplementary materials for

c-Myc-driven glycolysis polarizes functional regulatory B cells that trigger pathogenic inflammatory responses

Xu-Yan Wang^1#^, Yuan Wei^1#^, Bo Hu^2#^, Yuan Liao^2#^, Xiaodong Wang^3#^, Wen-Hua Wan^1^, Chun-Xiang Huang^1^, Mahepali Mahabati^1^, Zheng-Yu Liu^1^, Jing-Rui Qu^1^, Xiao-Dan Chen^1^, Dong-Ping Chen^1^, Dong-Ming Kuang^1^*, Xue-Hao Wang^4^* and Yun Chen^5,6^*

^1^MOE Key Laboratory of Gene Function and Regulation, Guangdong Province Key Laboratory of Pharmaceutical Functional Genes, School of Life Sciences, Sun Yat-sen University, Guangzhou, China; ^2^Department of Laboratory Medicine, the Third Affiliated Hospital of Sun Yat-sen University, Guangzhou, China; ^3^School of Pharmaceutical Sciences, Shenzhen University Health Science Center, Shenzhen, China; ^4^Hepatobiliary Center, The First Affiliated Hospital of Nanjing Medical University, Key Laboratory of Liver Transplantation, Chinese Academy of Medical Sciences, NHC Key Laboratory of Living Donor Liver Transplantation (Nanjing Medical University), Nanjing, China; ^5^Department of Immunology, Key Laboratory of Human Functional Genomics of Jiangsu Province, Nanjing Medical University, Nanjing, China and ^6^Jiangsu Key Lab of Cancer Biomarkers, Prevention and Treatment, Collaborative Innovation Center for Cancer Personalized Medicine, Nanjing Medical University, Nanjing, China

Correspondence: Yun Chen (chenyun@njmu.edu.cn) or Xue-Hao Wang (wangxh@njmu.edu.cn) or Dong-Ming Kuang (kdming@mail.sysu.edu.cn)

These authors contributed equally: Xu-Yan Wang, Yuan Wei, Bo Hu, Yuan Liao, Xiaodong Wang

**This file includes:**

**Figures S1 to S5**

**Tables S1 to S6**

**Fig. S1.**


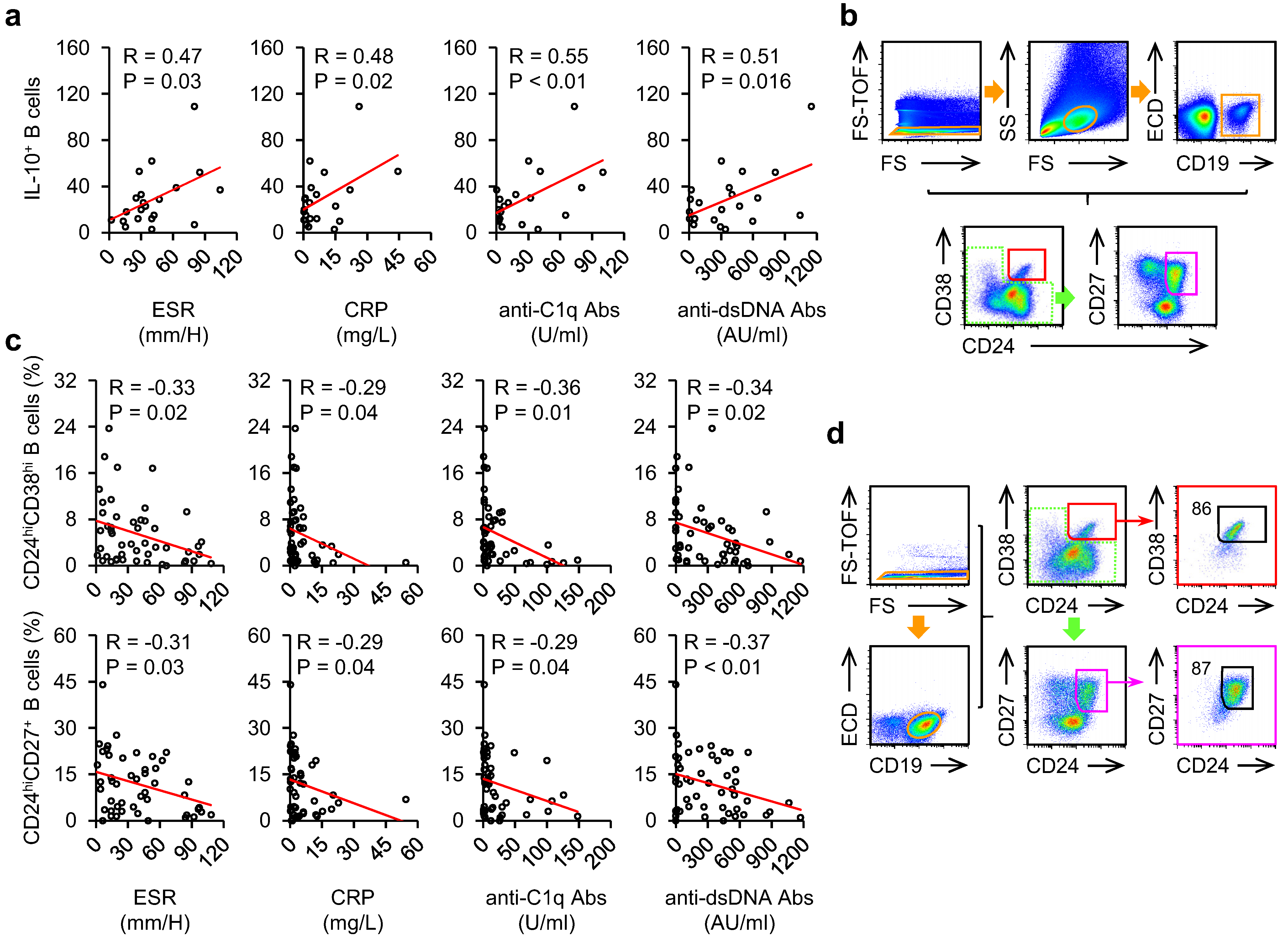


**Fig. S1 Clinical relevance of B cell subsets in the blood of patients with SLE. a** Associations of circulating IL-10^+^ B cells with the pathological parameters in SLE (*n* = 21). ESR, erythrocyte sedimentation rate; CRP, C-reactive protein; C1q, complement C1q; dsDNA, double-stranded DNA. **b** Gating strategy for FACS analysis. Representative plots of cells isolated from SLE patients’ blood showing that mononuclear cells were first gated for singlets (FS-TOF vs. FS) and lymphocytes (SS vs. FS), then CD19^+^ B cells (CD19 vs. ECD (unstained control determine auto-fluorescence); orange arrows). Thereafter, CD19^+^ B cells were further divided into CD24^hi^CD38^hi^ and CD24^hi^CD27^+^ subsets. **c** Associations of circulating CD24^hi^CD38^hi^ and CD24^hi^CD27^+^ B cells with the pathological parameters in SLE (*n* = 49). **d** Gating strategy for FACS sorting. CD19^+^ B cells were sorted with a MACS column purification system as described in Methods. CD24^hi^CD38^hi^ and CD24^hi^CD27^+^ subsets were further sorted according to patterns of CD24, CD27 and CD38 expression. Data are presented as at least four independent experiments. Pearson’s correlation analysis for **a** and **c**.

**Fig. S2.**


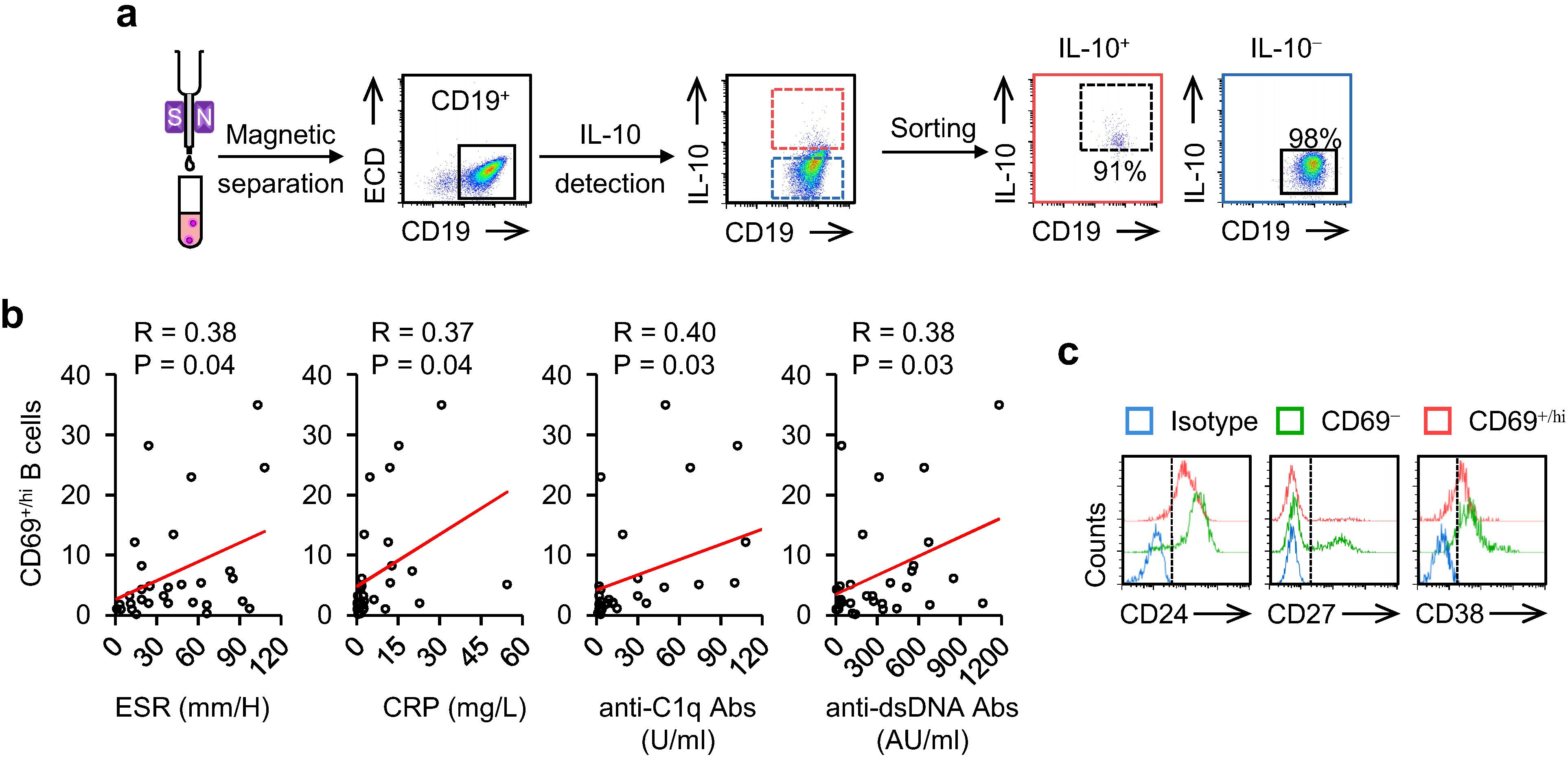


**Fig. S2 Phenotypic characteristics and pathological relevance of functional B_reg_ cells in SLE. a** IL-10^+^ and IL-10^−^ B cells from untreated SLE patients’ blood were sorted by Human IL-10 Secretion Assay-Detection Kit. Sorting efficacy was determined. **b** Associations of circulating CD69^+/hi^ B cells with the pathological parameters in SLE (*n* = 30). **c** FACS analysis of CD24, CD27, and CD38 expression on CD69^+/hi^ B cells from untreated SLE patients’ blood (*n* = 5). Data are presented as four independent experiments. Pearson’s correlation analysis for **b**.

**Fig. S3.**

**
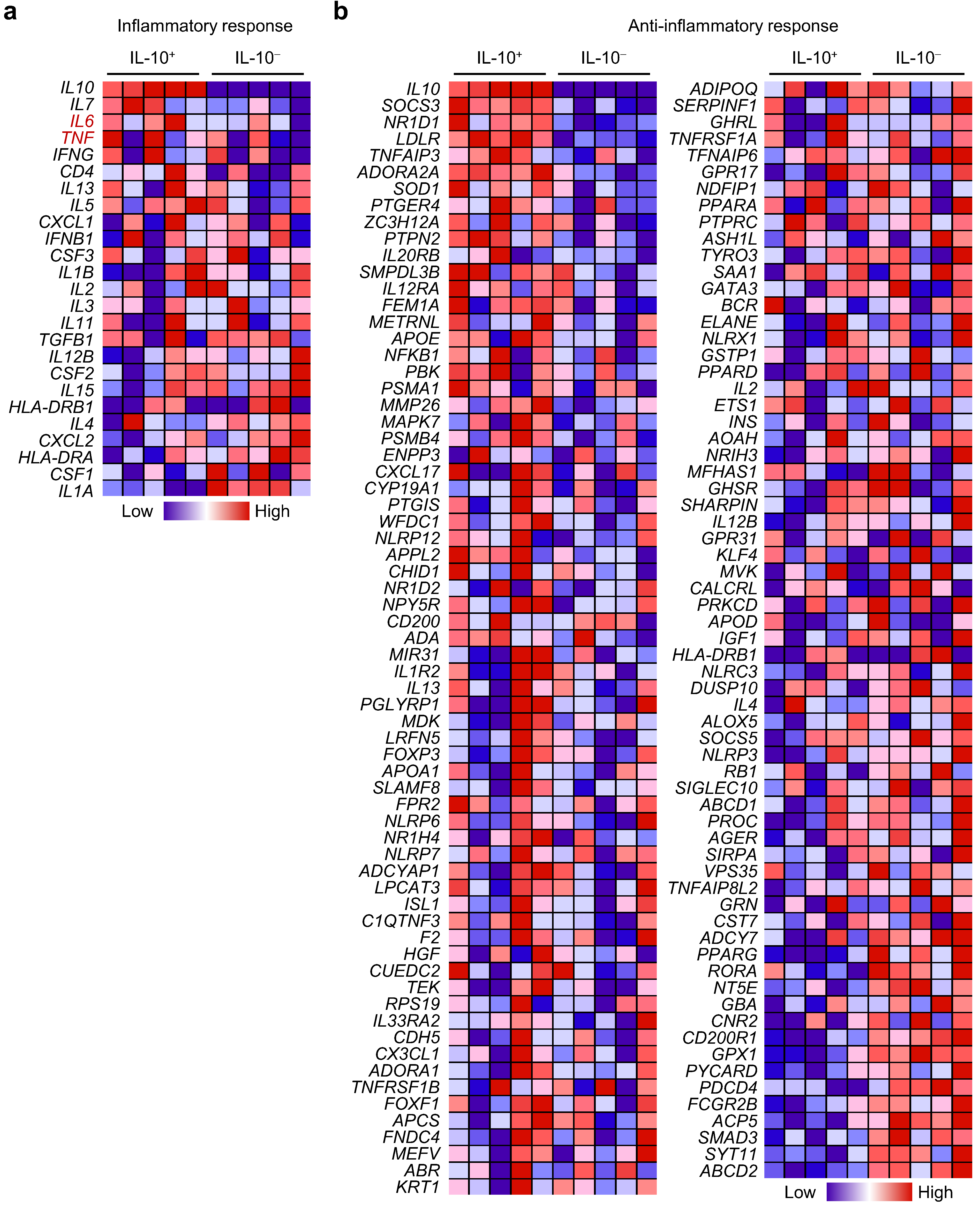
**

**Fig. S3 IL-10^+^ B cells display an inflammatory phenotype. a**, **b** Heat map showing expression of genes associated with inflammatory processes (WP530) (**a**) and anti-inflammatory processes (GO: 0050728) (**b**) in IL-10^+^ B cells and IL-10^−^ B cells (*n* = 5).

**Fig. S4.**


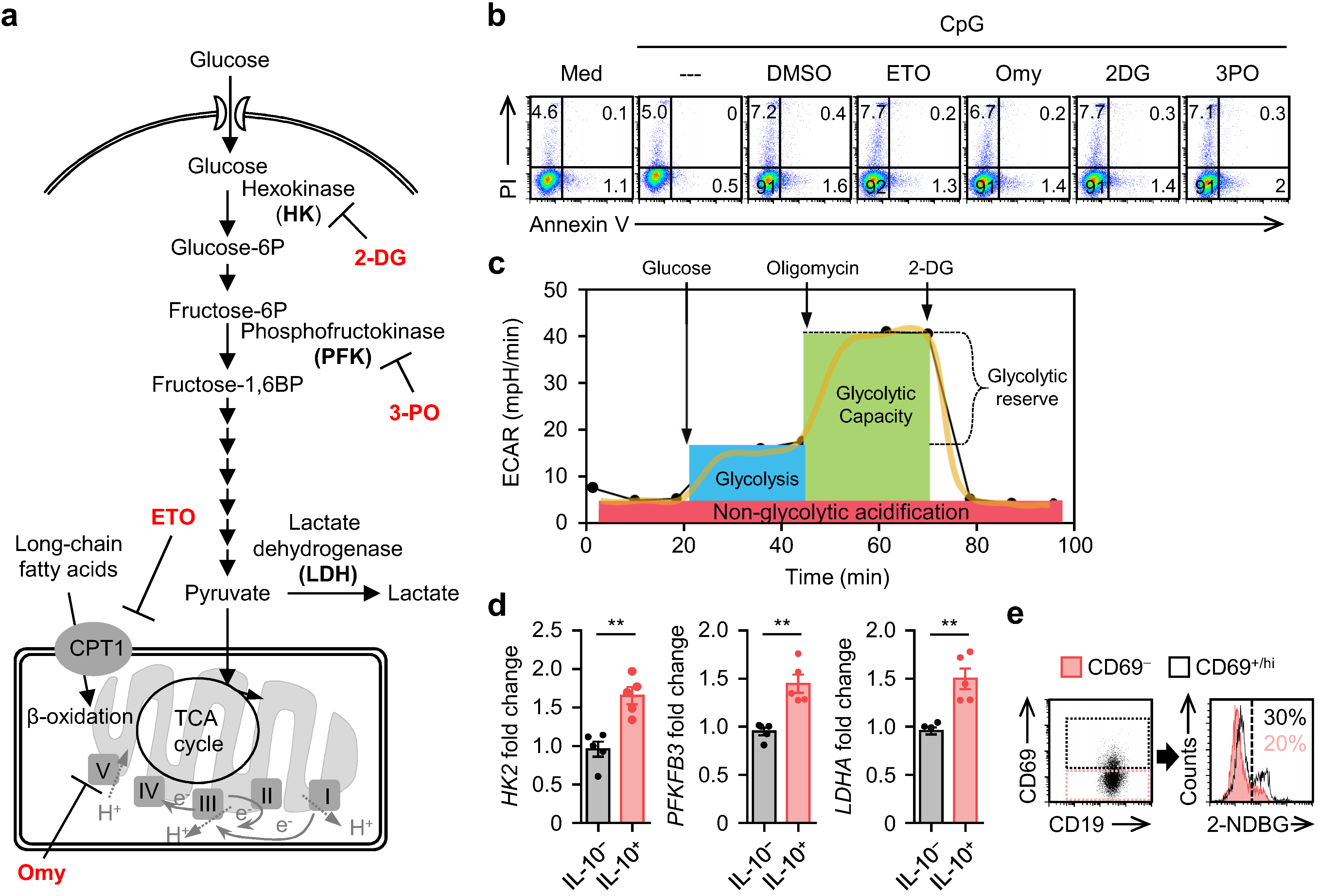


**Fig. S4 Glycolysis is required for the differentiation of inflammatory B_reg_ cells. a** Graphic showing key rate-limiting enzymes during glycolysis, oxidative phosphorylation, and fatty acid metabolism, as well as their inhibitors. **b** Purified total B cells from healthy donors were left untreated or were pretreated with DMSO, Etomoxir (ETO), Oligomycin (Omy), 2-Deoxyglucose (2-DG), or 3-PO. Thereafter, the cells were cultured in the presence or absence of CPG-DNA for 12 h. Apoptosis of cells were determined by FACS (*n* = 3). **c** Graphic showing glycolysis stress test profile of the key parameters of glycolytic function. **d** Expression of *HK2*, *PFKFB3*, and *LDHA* in IL-10^−^ and IL-10^+^ B cells isolated from untreated SLE patients were detected by real-time PCR (*n* = 5). **e** The abilities of glucose incorporation (2-NBDG^+^) by circulating CD69^−^ and CD69^+/hi^ B cells isolated from SLE patients were analyzed by FACS (*n* = 8). Data are presented as three independent experiments. Significance was determined with Student’s *t* test (**d**). ***p* < 0.01.

**Fig. S5.**





**Fig. S5 Signals that participate in glycolysis-triggered inflammatory B_reg_ cells. a** Purified total B cells from healthy donors were left untreated or exposed to CPG-DNA. Activation of mTOR and HIF-1α signals were determined by immunoblotting (*n* = 3). **b**-**e** Purified total B cells from healthy donors were left untreated or were pretreated with DMSO, inhibitors against mTOR or HIF-1α. Thereafter, the cells were cultured in the presence or absence of CPG-DNA. Extracellular acidification rate (ECAR) (**b**), expression of glycolytic enzymes (**c**, **d**), and cytokine production (**e**) were detected by seahorse analyzer, real-time PCR, immunoblotting and ELISA, respectively (each *n* = 5). **f, g** GSEA of c-Myc signaling (M5928) in IL-10^+^ B cells versus IL-10^−^ B cells (**f**). Heat map showing the expression of genes associated with c-Myc signaling that among the top 2000 upregulated genes in IL-10^+^ B cells (**g**). NES, normalized enrichment score. **h, i** Purified IL-10^+^ B cells from untreated SLE patients were left untreated or were pretreated with DMSO or inhibitor against c-Myc signal. Glycolytic enzyme expression (**h**) and cytokine production (**i**) were detected (*n* = 5). Data are presented as means ± SEM of three independent experiments (one-way ANOVA test for **b**, **c**, **e**, **h**, **i**). **p* < 0.05, ****p* < 0.001.

Table S1. Clinical characteristics of the SLE patients

| **Patients characteristics** | **Cohort 1**  **(untreated)** | **Cohort 2**  **(untreated)** | **Cohort 3**  **(clinical remission)** | **Cohort 4**  **(complete remission)** |
| --- | --- | --- | --- | --- |
| No. of patients | 71 | 219 | 15 | 12 |
| Age, years (median, range) | 30, 5-72 | 30, 9-83 | 34, 10-56 | 44, 27 -62 |
| Sex (male/female) | 8/63 | 29/190 | 3/12 | 1/11 |
| ESR, mm/H（≤20/>20） | 24/47 | 96/123 | 7/8 | NA |
| CRP, mg/L（≤6/>6） | 48/23 | 145/74 | 4/11 | NA |
| anti-C1q, U/ml（≤20/>20） | 48/23 | 150/69 | 8/7 | NA |
| anti-dsDNA, AU/ml（≤200/>200） | 28/43 | 106/113 | 6/9 | NA |
| SLEDAI (median, range) | 8, 3-19 | 9, 3-20 | NA | NA |

Abbreviations: ESR, erythrocyte sedimentation rate; CRP, C-reactive protein; C1q, complement C1q; dsDNA, double-stranded DNA; SLEDAI, systemic lupus erythematosus disease activity index.

Table S2. Characteristics of patients used in clinical relevance analyses

| **No.** | **Gender** | **Age**  **(years)** | **ESR**  **(mm/H)** | **CRP**  **(mg/L)** | **anti-C1q Ab**  **(U/ml)** | **anti-dsDNA Ab**  **(AU/ml)** | **SLEDAI** |
| --- | --- | --- | --- | --- | --- | --- | --- |
| 1 | F | 34 | 1 | 10.4 | 3 | 19 | 6 |
| 2 | F | 58 | 66 | 2.3 | 6 | 679 | 7 |
| 3 | F | 40 | 48 | 54.5 | 74 | 103 | 11 |
| 4 | F | 29 | 38 | 0.2 | 8 | 103 | 11 |
| 5 | F | 39 | 42 | 2.7 | 19 | 193 | 15 |
| 6 | M | 22 | 38 | 1.3 | 49 | 510 | 8 |
| 7 | F | 20 | 83 | 20.2 | 126 | 550 | 11 |
| 8 | F | 42 | 19 | 12.8 | 148 | 560 | 10 |
| 9 | M | 30 | 14 | 11.4 | 108 | 670 | 10 |
| 10 | F | 19 | 15 | 0.5 | 3 | 138 | 9 |
| 11 | F | 48 | 85 | 1.9 | 30 | 850 | 14 |
| 12 | F | 5 | 24 | 22.8 | 36 | 1063 | 8 |
| 13 | F | 46 | 35 | 0.4 | 30 | 264 | 8 |
| 14 | F | 12 | 11 | 0.2 | 12 | 333 | 8 |
| 15 | F | 30 | 3 | 0.5 | 2 | 23 | 4 |
| 16 | F | 34 | 53 | 2.9 | 6 | 0 | 5 |
| 17 | F | 22 | 108 | 12.1 | 68 | 638 | 16 |
| 18 | F | 34 | 24 | 15.3 | 102 | 39 | 13 |
| 19 | F | 35 | 62 | 12.2 | 100 | 393 | 9 |
| 20 | F | 53 | 10 | 2.5 | 2 | 223 | 9 |
| 21 | M | 17 | 12 | 2.5 | 2 | 340 | 3 |
| 22 | F | 26 | 19 | 6.3 | 9 | 33 | 7 |
| 23 | F | 17 | 25 | 1.9 | 2 | 27 | 5 |
| 24 | F | 22 | 56 | 0.5 | 12 | 41 | 11 |
| 25 | F | 14 | 66 | 1.6 | 1 | 119 | 10 |
| 26 | F | 27 | 97 | 2.6 | 15 | 441 | 10 |
| 27 | F | 45 | 4 | 0.3 | 4 | 0 | 6 |
| 28 | M | 25 | 19 | 1.5 | 3 | 5 | 5 |
| 29 | F | 59 | 92 | 2.8 | 3 | 272 | 9 |
| 30 | F | 18 | 55 | 4.8 | 3 | 310 | 7 |
| 31 | F | 50 | 14 | 4.4 | 2 | 0 | 9 |
| 32 | F | 49 | 84 | 3.6 | 27 | 1173 | 14 |
| 33 | F | 30 | 46 | 1.9 | 16 | 430 | 12 |
| 34 | F | 41 | 49 | 0.7 | 12 | 446 | 13 |
| 35 | F | 27 | 39 | 6.3 | 1 | 497 | 8 |
| 36 | F | 44 | 14 | 1.9 | 2 | 0 | 3 |
| 37 | F | 25 | 20 | 1.9 | 2 | 122 | 15 |
| 38 | F | 56 | 46 | 0.6 | 2 | 563 | 3 |
| 39 | F | 9 | 4 | 0.1 | 5 | 530 | 8 |
| 40 | F | 24 | 6 | 0.3 | 2 | 0 | 3 |

| 41 | F | 34 | 87 | 0.2 | 7 | 563 | 8 |
| --- | --- | --- | --- | --- | --- | --- | --- |
| 42 | M | 23 | 8 | 0.6 | 1 | 11 | 7 |
| 43 | F | 28 | 14 | 0.3 | 3 | 0 | 6 |
| 44 | F | 59 | 99 | 6.2 | 1 | 884 | 15 |
| 45 | F | 72 | 46 | 5 | 8 | 239 | 4 |
| 46 | F | 24 | 6 | 1.1 | 26 | 0 | 8 |
| 47 | F | 27 | 96 | 19.6 | 13 | 574 | 12 |
| 48 | F | 20 | 33 | 5.1 | 12 | 484 | 7 |
| 49 | F | 18 | 6 | 0.2 | 2 | 455 | 6 |
| 50 | F | 34 | 103 | 30.8 | 50 | 1287 | 18 |
| 51 | F | 21 | 32 | 2.8 | 11 | 92 | 3 |
| 52 | M | 15 | 15 | 2.3 | 5 | 293 | 4 |
| 53 | M | 22 | 30 | 6.1 | 18 | 402 | 10 |
| 54 | F | 41 | 40 | 2.9 | 30 | 302 | 13 |
| 55 | F | 20 | 13 | 17 | 2 | 595 | 6 |
| 56 | F | 14 | 40 | 14.5 | 39 | 340 | 9 |
| 57 | F | 37 | 47 | 0.6 | 3 | 9 | 12 |
| 58 | F | 26 | 28 | 44.4 | 41 | 500 | 7 |
| 59 | F | 16 | 25 | 0.7 | 32 | 642 | 16 |
| 60 | F | 36 | 40 | 3.1 | 3 | 52 | 6 |
| 61 | F | 56 | 30 | 0.3 | 2 | 305 | 15 |
| 62 | F | 23 | 85 | 9.8 | 100 | 808 | 9 |
| 63 | F | 41 | 2 | 0.2 | 2 | 235 | 5 |
| 64 | F | 13 | 42 | 1.1 | 65 | 1042 | 8 |
| 65 | F | 39 | 104 | 21.8 | 0 | 17 | 12 |
| 66 | F | 37 | 63 | 3.6 | 80 | 374 | 14 |
| 67 | F | 23 | 80 | 26.1 | 73 | 1148 | 19 |
| 68 | F | 42 | 80 | 1.4 | 24 | 43 | 10 |
| 69 | F | 62 | 34 | 15 | 7 | 473 | 7 |
| 70 | M | 52 | 16 | 0.2 | 4 | 0 | 5 |
| 71 | F | 60 | 27 | 6.2 | 2 | 0 | 5 |

Table S3. Antibodies for immunoblotting

| **Antibody** | **Supplier** | **Identifier** |
| --- | --- | --- |
| Phospho-mTOR (Ser2448) (D9C2) XP Antibody | Cell Signaling Technology | Cat# 5536; RRID:AB_10691552 |
| mTOR (7C10) Antibody | Cell Signaling Technology | Cat# 2983; RRID:AB_2105622 |
| Mouse Anti-Human HIF-1α | BD Biosciences | Cat# 610958; RRID:AB_398271 |
| Rabbit Anti-human c-MYC | Cell Signaling Technology | Cat# 9402; RRID:AB_2151827 |
| Rabbit Anti-human Hexokinase II (C64G5) | Cell Signaling Technology | Cat# 2867; RRID:AB_2232946 |
| Rabbit Anti-Human PFKFB3 | Proteintech | Cat# 13763-1-AP; RRID:AB_2162854 |
| Rabbit Anti-Human LDHA Antibody | Proteintech | Cat# 19987-1-AP; RRID:AB_10646429 |
| Phospho-NF-κB p65 (Ser536) (93H1) Antibody | Cell Signaling Technology | Cat# 3033; RRID:AB_331284 |
| NF-kappaB p65 (D14E12) XP antibody | Cell Signaling Technology | Cat# 8242; RRID:AB_10859369 |
| Phospho-NF-kappaB2 p100 (Ser866/870) Antibody | Cell Signaling Technology | Cat# 4810; RRID:AB_659925 |
| NF-κB2 p100/p52 Antibody | Cell Signaling Technology | Cat# 4882; RRID:AB_10695537 |
| Phospho-p38 (Thr180/Tyr182) (D3F9) XP Antibody | Cell Signaling Technology | Cat# 4511; RRID:AB_2139682 |
| p38 (D13E1) XP Antibody | Cell Signaling Technology | Cat# 8690; RRID:AB_10999090 |
| Phospho-Erk1/2 (Thr202/Tyr204) Antibody | Cell Signaling Technology | Cat# 4370; RRID:AB_2315112 |
| Erk1/2 (137F5) Antibody | Cell Signaling Technology | Cat# 4695; RRID:AB_390779 |
| Phospho-JNK (Thr183/Tyr185) (81E11) Antibody | Cell Signaling Technology | Cat# 4668; RRID:AB_823588 |
| SAPK/JNK Antibody | Cell Signaling Technology | Cat# 9252; RRID:AB_2250373 |
| β-Actin (ACTB) Antibody (8H10) | Origene | Cat# TA310155; RRID:AB_10691552 |

Table S4. Fluorochrome-conjugated antibodies used in flow cytometry

| **Antibody** | **Supplier** | **Catalogue** | **RRID** |
| --- | --- | --- | --- |
| Anti-CCR7 Antibody, AF 700, Clone 150503 | R&D Systems | FAB197N | AB_10995036 |
| Anti-CD138 Antibody, AF647, Clone MI15 | BD Biosciences | 562097 | AB_10895974 |
| Anti-CD19 Antibody, PE-CY7, Clone J4.119 | Beckman Coulter | IM3628U | AB_10638575 |
| Anti-CD19 Antibody, BV421 | BioLegend | 302234 | AB_11142678 |
| Anti-CD20 Antibody, FITC, Clone B9E9 | Beckman Coulter | IM1455U | AB_131018 |
| Anti-CD20 Antibody, eFluor 450, Clone 2H7 | eBioscience | 48-0209-42 | AB_1633384 |
| Anti-CD24 Antibody, APC-eFluor 780, Clone (eBioSN3 (SN3 A5-2H10)) | eBioscience | 47-0247-42 | AB_10735091 |
| Anti-CD25 Antibody, PE, Clone BC96 | eBioscience | 12-0259-42 | AB_1659682 |
| Anti-CD27 Antibody, BV421, Clone HIT2 | BD Biosciences | 562513 | AB_11153497 |
| Anti-CD210 (IL-10 R) Antibody, APC | BioLegend | 308812 | AB_2564593 |
| Anti-CD38 Antibody, FITC, Clone HIT2 | BD Biosciences | 560982 | AB_2033957 |
| Anti-CD4 Antibody, BV421, Clone OKT4 | BioLegend | 317434 | AB_2562134 |
| Anti-CD4 Antibody, PE-CY7 | Beckman Coulter | 6607101 | AB_10641616 |
| Anti-CD45RA Antibody, ef 450, Clone HI100 | eBioscience | 48-0458-42 | AB_1272059 |
| Anti-CD69 Antibody (FN50), APC | BD Biosciences | 555533 | AB_398602 |
| Anti-CD69 Antibody (FN50), PE | Beckman Coulter | IM1943U | AB_2801272 |
| Anti-CD8 Antibody, PE, Clone B9.11 | Beckman Coulter | IM0452U | AB_131202 |
| Anti-CD80 Antibody, PE, Clone 2D10.4 | eBioscience | 12-0809-42 | AB_1311209 |
| Anti-CD86 Antibody, PE, Clone HA5.2B7 | Beckman Coulter | IM2729U |  |
| Anti-HLA-DR Antibody, PE-CF594, Clone G46-6 | BD Biosciences | 562304 | AB_11154415 |
| Anti-IFN-γ Antibody, ef450, Clone 4S.B3 | eBioscience | 48-7319-42 | AB_2043866 |
| Anti-IgA Antibody, PE | Southern Biotech | 2050-09 | AB_2795707 |
| Anti-IgD Antibody, AF-700, Clone IA6-2 | BD Biosciences | 561302 | AB_10646035 |
| Anti-IgG Fc Antibody, AF647 | BioLegend | 409319 | AB_2563329 |
| Anti-IgG Antibody, APC-H7, Clone G18-145 | BD Biosciences | 561297 | AB_10611877 |
| Anti-IgM Antibody, AF647 | BioLegend | 314535 | AB_2566612 |
| Anti-IgM Antibody, FITC, Clone G20-127 | BD Biosciences | 555782 | AB_396117 |
| Anti-IL-6 Antibody, PE, Clone MQ2-13A5 | eBioscience | 12-7069-82 | AB_466168 |
| Anti-IL-10 Antibody, APC, Clone JES3-19F1 | BD Biosciences | 554707 | AB_398582 |
| Anti-IL-17A Antibody, FITC, Clone TC11-18H10 | BD Biosciences | 559502 | AB_397256 |
| Anti-IL-22 Antibody, PE, Clone 22URTI | eBioscience | 12-7229-42 | AB_1834463 |
| Anti-PD-L1 Antibody, PE, Clone MIH1 | eBioscience | 12-5983-42 | AB_11042286 |
| Anti-TGF-β Antibody, PE, Clone 27232 | R&D Systems | FAB2463P | AB_884505 |
| Anti-TNF-α Antibody, ef 450, Clone MAb11 | eBioscience | 48-7349-42 | AB_2043889 |
| Mouse IgG1 κ Isotype, FITC, Clone MOPC-21 | BD Biosciences | 555748 | AB_396090 |
| Mouse IgG1 κ Isotype, PE, Clone P3.6.2.8.1 | eBioscience | 12-4714-82 | AB_470060 |
| Mouse IgG2b κ Isotype, PE, Clone eBMG2b | eBioscience | 12-4732-81 | AB_763659 |
| Rat IgG2a κ Isotype, APC, Clone RTK2758 | BioLegend | 400512 | AB_2814702 |
| Annexin V-FITC Reagent | BioVision | 1001 | AB_10995036 |

Table S5. Recombinant proteins, peptides, chemicals, and critical commercial assays

| **Name** | **Supplier** | **Identifier** | |
| --- | --- | --- | --- |
| Recombinant proteins, peptides | | |  |
| Anti-human IgM Antibody | Jackson ImmunoResearch | Cat# 109-006-129; RRID: AB_2337553 | |
| Human CD40/TNFRSF5 Antibody | R&D Systems | Cat# AF632; RRID:AB_355490 | |
| Human IL-10 Antibody, Clone 23738 | R&D Systems | Cat# MAB217; RRID:AB_358064 | |
| Human IL-6 Antibody, Clone 6708 | R&D Systems | Cat# MAB206; RRID:AB_2127617 | |
| Human TNF-alpha Antibody, Clone 28401 | R&D Systems | Cat# MAB610; RRID:AB_2203945 | |
| CD3 monoclonal Antibody, Clone UCHT1 | eBioscience | Cat# 16-003885; RRID: AB_468857D | |
| CD28 Monoclonal Antibody, Clone CD28.2 | eBioscience | Cat# 16-028985; RRID: AB_468927 | |
| Mouse IgG1 isotype control, Clone 11711 | R&D Systems | Cat# MAB002; RRID:AB_357344 | |
| Recombinant Human IL-2 | R&D Systems | Cat# 202-IL-010 | |
| **Chemicals** | | |  |
| Lipopolysaccharides | Sigma | Cat# L2880 | |
| CPG oligodeoxynucleotides (ODNs) | InvivoGen | Cat# tlrl-2395 | |
| Etomoxir | Sigma | Cat# 236020; CAS: 828934-41-4 | |
| Oligomycin | Sigma | Cat# 495455; CAS: 1404-19-9 | |
| Propidium iodide | Sigma | Cat# P4170; CAS: 25535-16-4 | |
| 2-DG | Sigma | Cat# D8375; CAS: 154-17-6 | |
| 3-PO | Merck Millipore | Cat# 525330; CAS: 18550-98-6 | |
| 2-NBDG | Sigma | Cat# 72987; CAS: 186689-07-6 | |
| Rapamycin | Sigma | Cat# V900930; CAS: 53123-88-9 | |
| Echinomycin | Cayman | Cat# 11049; CAS: 512-64-1 | |
| (+)-JQ1 | APExBio | Cat# A1910; CAS: 1268524-70-4 | |
| Bay 11-7082 | Sigma | Cat# B5556; CAS: 19542-67-7 | |
| Amgen 16 | Sigma | Cat# SML2457; CAS: 1202761-92-9 | |
| U 0126 | Sigma | Cat# U120; CAS: 109511-58-2 | |
| SB 203580 | Sigma | Cat# S8307; CAS: 152121-47-6 | |
| SP 600125 | Sigma | Cat# S5567; CAS: 129-56-6 | |
| Critical commercial assays | | |  |
| CD19 MicroBeads | Miltenyi Biotec | Cat# 130-050-301 | |
| Human IL-10 ELISPOT Pair | BD Biosciences | Cat# 551883 | |
| IL-10 Secretion Assay-Detection Kit, human | Miltenyi Biotec | Cat# 130-090-434 | |
| Leukocyte Activation Cocktail | BD Biosciences | Cat# 550583 | |
| IntraPrep reagent | Beckman Coulter | Cat# A07803 | |
| TNF-α Human Uncoated ELISA Kit | eBioscience | Cat# 88-7347 | |
| IL-6 Human Uncoated ELISA Kit | eBioscience | Cat# 88-7066 | |
| IL-10 Human Uncoated ELISA Kit | eBioscience | Cat# 88-7106 | |
| 5X All-In-One RT MasterMix | Applied Biological Materials | Cat# G492 | |
| Hieff qPCR SYBR Green Master Mix | Yeasen Biotech | Cat# 11201ES03 | |
| XF Glycolysis Stress Test kit | Seahorse Bioscience | Cat# 102194-100 | |
| L-Lactate Assay Kit I -200 Assays | eton bioscience | Cat# 1200012002 | |

Table S6. Primers for real-time PCR

| **Gene** | **Forward** | **Reverse** |
| --- | --- | --- |
| *IL10* | CTTCGAGATCTCCGAGATGCCTTC | ATTCTTCACCTGCTCCACGGCCTT |
| *IL6* | TCAGCCCTGAGAAAGGAGACA | GATTTTCACCAGGCAAGTCTCC |
| *TNF* | AAGCCTGTAGCCCATGTTG | TGGTAGGAGACGGCGATG |
| *GLUT1* | CTTTGTGGCCTTCTTTGAAGTG | GACCACACAGTTGCTCCACATAC |
| *HK2* | GATTGTCCGTAACATTCTCATCG | CAGGCAGTCACTCTCAATCTGAG |
| *HK3* | CAGCAGCTCTTTGACCACATC | CGCAACAGACTCACGACATC |
| *PFKFB1* | CTCCATCTACCTTTGCCGACA | GCCCTGGGACTGAATGAAGTT |
| *PFKFB2* | CACCAATACAACCCGGGAGA | GCAGCAATGACATCAGGATCA |
| *PFKFB3* | CTCGCATCAACAGCTTTGAGG | TCAGTGTTTCCTGGAGGAGTC |
| *PFKFB4* | CCAACTGCCCAACTCTCATTG | GCGATACTGGCCAACATTGAA |
| *PFKL* | CTGTACTCATCAGAGGGCAAG | TGCCAGCATCTTCAGCATGAG |
| *ALDOA* | AGATGAGTCCACTGGGAGCAT | AGATGAGTCCACTGGGAGCAT |
| *ALDOC* | GGATGAGTCTGTAGGCAGCAT | GAGTGGTGGTTTCTCCATCAG |
| *GAPDH* | GGAGTCAACGGATTTGGTCGT | TCTCGCTCCTGGAAGATGGT |
| *PGK1* | GGGTCGTTATGAGAGTCGACT | AGGTGGCTCATAAGGACTACC |
| *ENO1* | GCCCTGGTTAGCAAGAAACTG | TTCTCAACGGCACCAGCTTTG |
| *ENO2* | AACAGTGAAGCCTTGGAGCTG | TCCTCAATGGAGACCACAGGA |
| *PKM* | TCTGTACCATTGGCCCAGCTT | TGGCTGTGCGCACATTCTTGA |
| *LDHA* | GATTCCAGTGTGCCTGTATGG | CTACAGAGAGTCCAATAGCCC |
| *LDHB* | GCGTGTGCTATCAGCATTCTG | TTCTCTGCACCAGATTGAGCC |
| *PDK1* | GCTAGGCGTCTGTGTGATTTG | AACACCTCTGTTGGCATGGTG |
| *PDK3* | TCGCCGCTCTCCATCAAACAA | CTGAACCAATCCCACTGAAGG |
| *MYC* | CTGGGAAGAAGCCAGTTCAG | TGGGCCATAGGTTTTCAGAG |
| *ACTIN* | GGATGCAGAAGGAGATCACT | CGATCCACACGGAGTACTTG |
